# Nickel Metalloregulators and Chaperones

**DOI:** 10.3390/inorganics7080104

**Published:** 2019-08-19

**Authors:** Khadine Higgins

**Affiliations:** Department of Chemistry, Salve Regina University, Newport, RI 02840, USA

**Keywords:** nickel, metalloregulator, chaperone, [NiFe]-hydrogenase, urease

## Abstract

Nickel is essential for the survival of many pathogenic bacteria. *E. coli* and *H. pylori* require nickel for [NiFe]-hydrogenases. *H. pylori* also requires nickel for urease. At high concentrations nickel can be toxic to the cell, therefore, nickel concentrations are tightly regulated. Metalloregulators help to maintain nickel concentration in the cell by regulating the expression of the genes associated with nickel import and export. Nickel import into the cell, delivery of nickel to target proteins, and export of nickel from the cell is a very intricate and well-choreographed process. The delivery of nickel to [NiFe]-hydrogenase and urease is complex and involves several chaperones and accessory proteins. A combination of biochemical, crystallographic, and spectroscopic techniques has been utilized to study the structures of these proteins, as well as protein-protein interactions resulting in an expansion of our knowledge regarding how these proteins sense and bind nickel. In this review, recent advances in the field will be discussed, focusing on the metal site structures of nickel bound to metalloregulators and chaperones.

## 1. Introduction

Nickel, the twenty-eighth element in the periodic table, is an essential metal for the functioning of many proteins in archaea, bacteria, plants, and some eukaryotes [1]. It is a cofactor for at least eight enzymes including urease and [NiFe]-hydrogenase [1]. Many pathogenic bacteria require nickel for their survival and pathogenicity, including *Helicobacter pylori* (*H. pylori*), [2,3]. Despite being an essential metal, high concentrations of nickel can be toxic as nickel can bind to other metalloproteins and displace the cognate metals resulting in an inactive metalloprotein [4]. As such nickel concentrations in the cell need to be regulated. One way in which nickel concentrations are controlled in the cell is by utilizing metalloregulators. Nickel-responsive metalloregulators function by binding to nickel in a specific coordination environment resulting in the transcriptional repression, activation, or depression of genes associated with nickel export or import. To maintain nickel concentration in the cell, *E. coli* utilizes two metalloregulators, NikR and RcnR [5]. NikR controls the expression of the genes associated with the ATP binding cassette (ABC)-type nickel importer, NikABCDE [6], and RcnR, regulates the expression of the genes associated with the export proteins RcnAB [7,8].

**Figure 1.**
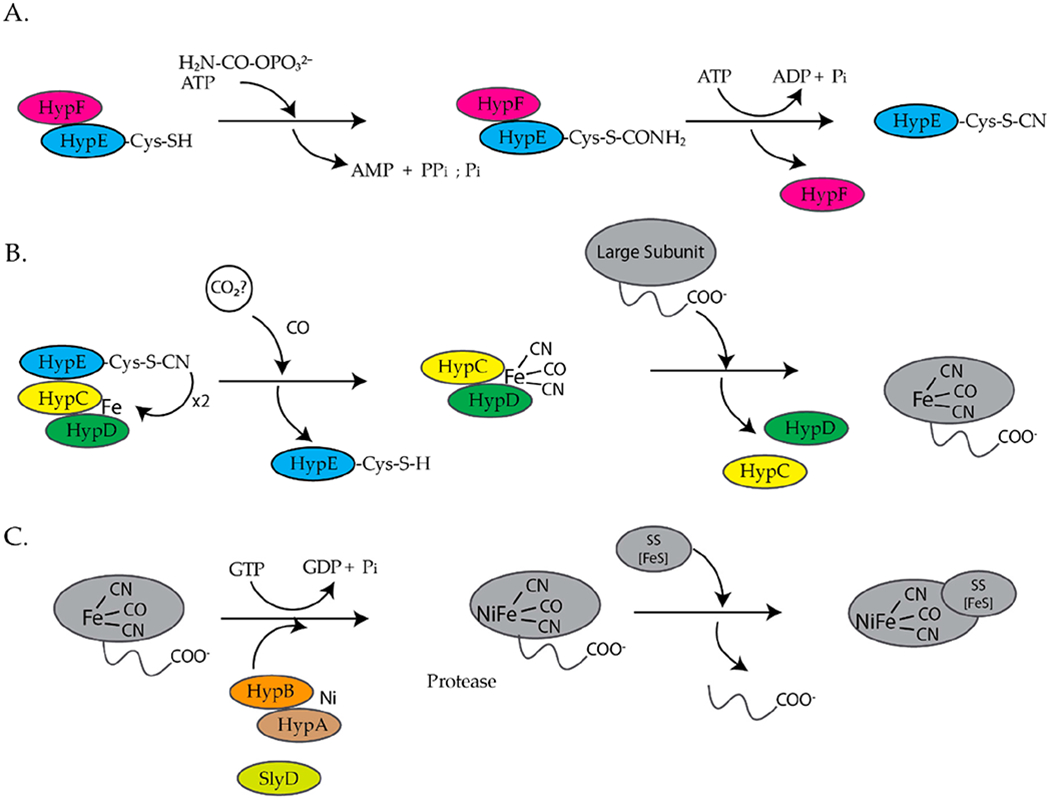
Model for the assembly [NiFe]-hydrogenase [12,13,17,20]. (**A**) HypE and HypF synthesize the cyanide ligands from carbamoylphosphate. (**B**) The assembly of the iron cofactor, Fe(CN)_2_CO, and delivery to the precursor large subunit of [NiFe}]-hydrogenase. (**C**) Nickel delivery and insertion into the large, precursor subunit of [NiFe]-hydrogenase and the peptide on the C-terminus of the large subunit is processed. The large subunit associates with the small subunit (SS) forming the mature [NiFe]-hydrogenase.

*E. coli* is a facultative anaerobe and under anaerobic conditions expresses four different [NiFe]-hydrogenases, hydrogenase 1,2,3, and 4, each associated with a specific metabolic pathway [9,10]. Hydrogenases are a group of metalloenzymes that catalyze the reversible oxidation of molecular hydrogen to protons and electrons [11]. [NiFe]-hydrogenases are composed of at least two subunits, a large and a small subunit [12,13]. The large subunit contains a complex NiFe(CN)_2_CO center at the active site and the small subunit contains up to three iron sulfur clusters [12,13]. The assembly of [NiFe]-hydrogenase 3 is dependent on the *hyp* (hydrogenase pleiotropy) genes *hypABCDEF* and *slyD* genes (Figure 1) [9,14–17]. For the assembly of hydrogenases 1 and 2, HypA and HypC are replaced by HybF and HybG [18]. HypE and HypF form a complex and synthesize the cyanide ligands from carbamoylphosphate (Figure 1A) [12,13,19]. HypE transfers the cyanide ligand to the HypC–HypD complex, which acts as a scaffold for the assembly and delivery of the iron cofactor, Fe(CN)_2_CO, to the precursor large subunit of [NiFe]-hydrogenase (Figure 1B) [12,13]. Finally, HypA, HypB, and SlyD insert nickel into the large, precursor subunit of [NiFe]-hydrogenase (Figure 1C) [12,13,17]. At least six review articles have been published within the last ten years that provide a more detailed description of the biosynthesis of hydrogenases [11–13,17,20,21].

**Figure 2.**
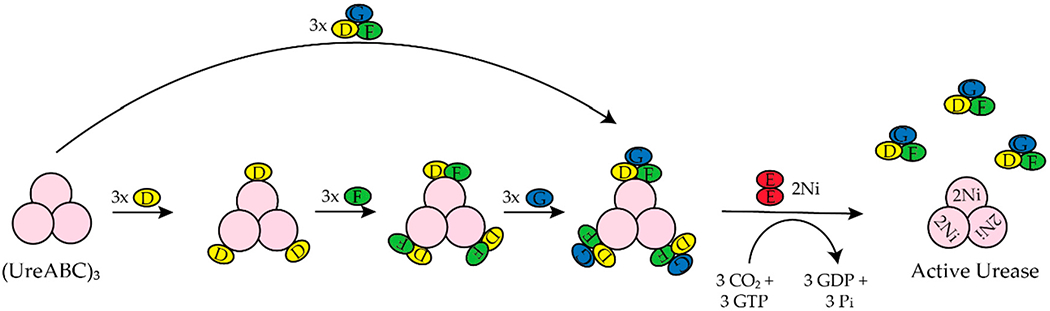
A simplified scheme of *K. aerogenes* urease activation [20,27,28]. The trimer of trimers (UreABC)_3_ sequentially binds UreD, UreF, UreG or the UreDFG complex. The isolated UreDFG complex contains two UreD, UreF, and UreG promoter but only one monomer is shown for simplicity. Lys 217 is carbamylated by CO_2_, GTP is hydrolyzed by UreG, and nickel is delivered by UreE to form the active enzyme and the UreDFG proteins are released.

*H. pylori* is a human pathogen that causes gastritis, peptic ulcers, and some types of gastric cancer [22–25]. Mouse studies determined that *H. pylori* requires [NiFe]-hydrogenase for efficient colonization of the gut [26]. *H. pylori also* requires urease to survive the acidic environment of the stomach [1]. Urease catalyzes the hydrolysis of urea to ammonia and carbamate, which reacts with water to yield a second molecule of ammonia and bicarbonate [1,27]. The ammonia produced is used to neutralize the cytoplasmic and periplasmic pH of the bacteria under acidic conditions [24]. The enzyme urease has been discussed extensively in the past ten years [1,20,27–30]. *H. Pylori* urease has two distinct types of subunits that combine to form a trimer of dimers [28]. Four of these trimer of dimers combine to form a super molecular complex of the form ((UreAB)_3_)_4_ [28]. The maturation of *H. pylori* urease is dependent on the accessory proteins ((UreAB)_3_)_4_ [28,29]. Unlike urease from *H. pylori,* urease from *Klebsiella aerogenes* (*K. aerogenes*) is composed of three subunits that form a trimer of trimers, (UreABC)_3_, structure [28]. In *K. aerogenes* the accessory proteins are UreDEFG, where UreD is homologous to (UreABC)_3_ in *H. pylori* [27,28]. UreI is a proton-gated urea channel that regulates urease activity as it permits urea entry into *H. pylori* [24]. UreI is located in the inner membrane of the bacteria; at pH 7.0 the channels are closed and at pH 5.0 the channels are open [24]. Figure 2 shows the current model for the activation of *K. aerogenes* urease [27,28].The exact role of UreD/UreH is unknown [28], UreE is the chaperone that delivers nickel to urease [3,29], and UreF has been suggested to be an activator of the GTPase activity of UreG. Formation of the active enzyme requires CO_2_ to carbamylate the lysine in the active site [27]. The active site of urease contains two nickel ions that are bridged by the oxygen atoms of a carbamylated lysine residue and a hydroxide ion [1]. Both nickel ions are bound by two different histidines [1]. One nickel ion is bound by aspartate and the other is bound by a water molecule [1]. Additionally, in *H. pylori* the [NiFe]-hydrogenase accessory proteins, HypA and HypB, are important for urease maturation [31–33]. Deletion of *hypA* or *hypB* resulted in a urease activity that was forty- and two hundred-fold lower than the wild-type strain, respectively [32]. Urease activity was restored with nickel supplementation in the growth medium [32].

A recent study by Jones et al. determined that urease is activated under acid shock conditions [34]. *H. pylori* cells were exposed to different pHs (6.8, 5.6, and 2.0) and the cytosolic pH of the bacteria was measured in the presence and absence of nickel sulfate [34]. Minor changes in the internal pH were observed when the bacteria was exposed to mild acidic conditions (pH 6.8 and 5.6) but when the bacteria was exposed to an external pH of 2.0 (pH of the stomach) the internal pH dropped to 5.1 initially and rapidly recovered to a pH of ~6.0 [34]. The drop in pH was less when nickel was supplemented in the *H. pylori* cultures [34]. It is thought that this apparent increase in buffering capacity is due to an increase in urease activation with increased nickel uptake [34,35]. Additionally, they determined that under acid shock conditions the following genes were regulated by *H. pylori* NikR: *ureA, nikR, amiE, amiF, arsR, ureG, nixA, frpB2* [34]. These results demonstrate that *H. pylori* NikR is responsive to pH changes in the cytosol.

In this review, several nickel metalloregulators and chaperones from various organisms will be discussed. We will focus on how nickel binding to metalloregulators results in conformational changes in the protein that influence DNA binding. We will also examine the metal site structures, binding affinities, and the associated conformational changes that accompany nickel binding to these proteins. The roles that the nickel chaperone proteins play in the maturation of [NiFe]-hydrogenase and urease as well as how these proteins interact with other chaperone proteins and the target proteins will be discussed.

## 2. Nickel-Responsive Metalloregulators

Metalloregulators can be divided into two groups depending on whether cognate metal ion binding results in the downregulation of metal uptake systems or the upregulation of metal efflux/sequestration systems [36]. The nickel-responsive metalloregulators NikR and Nur belong to the first group as nickel binding to the protein results in conformational changes that favors DNA binding leading to the repression of the genes encoding proteins associated with import. RcnR, InrS, DmeR, and NmtR belong to the second group as nickel binding to the protein causes conformational changes in the protein that disfavors DNA binding allowing for the expression of the genes encoding proteins associated with export. Nickel-responsive metalloregulators have been characterized in five of the seven major families of transcriptional metalloregulators in prokaryotes [36]. These include NikR (NikR family), RcnR (RcnR/CsoR family), InrS (RcnR/CsoR family), DmeR (RcnR/CsoR family), NmtR (ArsR family), KmtR (ArsR family), NimR (MerR family), Nur (Fur family). These metalloregulators have been reviewed at least three times in the last ten years [5,20,37]. The respective K_d_s and the pH at which the K_d_ was determined, the coordination number (CN #), and the ligands used to coordinate the metal ion are included in Table S1.

### 2.1. NikR

NikR is a Ni(II)-responsive metalloregulator that inhibits the expression of the genes associated with Ni(II) import in the presence of excess Ni(II) [6]. In *E. coli,* NikR controls the expression of the *nik* operon that encodes the *E. coli* Ni(II) importer NikABCDE [6]. Ni(II)-NikR binds to a palindromic operator sequence within the *nikABCDE* promoter, GTATGA-N_16_-TCATAC, with nanomolar affinity [38–40] Like *E. coli, Brucella abortus* also has a putative nickel importer NikABCDE and NikR controls the expression of *nikA* [41]. *H. pylori* NikR controls the expression of genes encoding the Ni(II) import protein NixA as well as the urease structural genes (*urea* [42]-*ureB*), Ni(II) uptake factors (*fecA3, frpB4*, and *exbB/exbD*), Ni(II) storage genes (*hpn* and *hpn*-like) and genes associated with iron uptake (*fur* and *pfr*) [34,43]. A recent study by Vannini et al. determined that NikR controlled the expression of the genes encoding two outer membrane proteins that are predicted to be metal ion transporters (*hopV* and *hopW*), and an outer membrane-absorbed protein (*hcpC*) [44]. Additionally, it was determined that NikR regulated the expression of genes predicted to be associated with toxin-antitoxin systems (*dvnA, mccB*), a gene predicted to be associated with metabolism (*phbA*), and a putative component of an ABC transporter (*fecD*) as well as non-coding RNAs (nrr1, nrr2 and isoB) [44].

**Figure 3.**
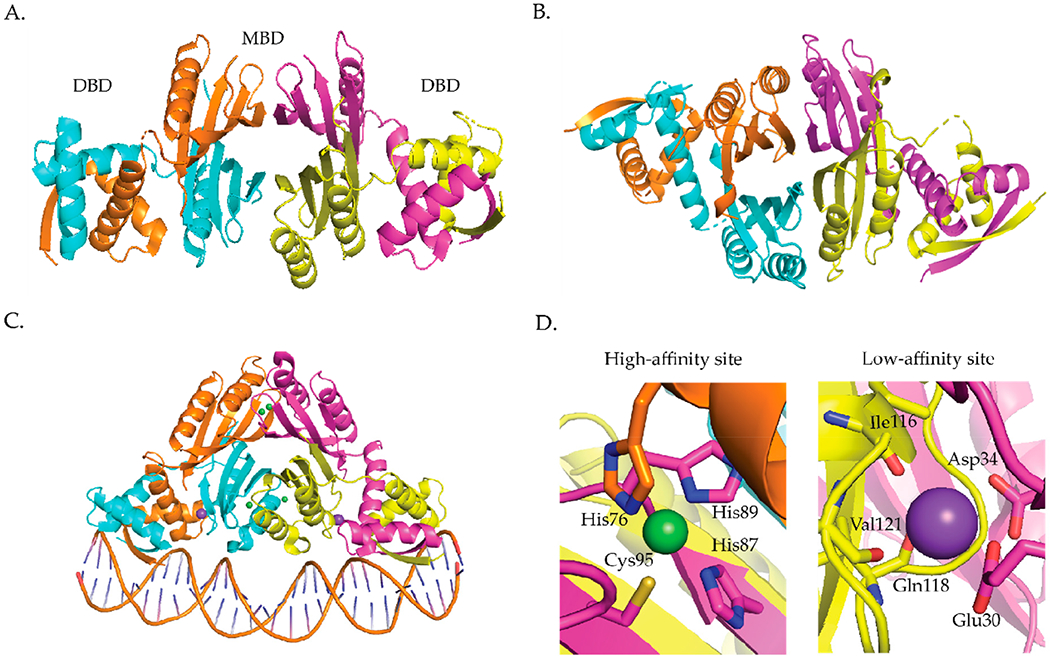
NikR proteins from *E. coli*, and *H. pylori* showing different conformations. The NikR monomers are colored in orange, blue, purple, and yellow, the nickel ions are shown as green spheres and the potassium ions are shown as purple spheres. (**A**) Apo *E. coli* NikR (PDB ID 1Q5V) [45] in an open conformation where the DBDS are placed linearly on each side of MBD. (**B**) *H. pylori* Ni(II)-NikR (PDB ID 2CAD) [54] depicting a closed trans-conformation. (**C**) *E. coli* NikR bound to DNA, nickel ions and potassium ions (PDB 2HZV) [46]. (**D**) Closeup of the high- and low-affinity sites where the nickel ion and potassium ion adopt square planar and octahedral geometries, respectively. (Figure adapted with permission from Higgins, K.A.; Giedroc, D.P. Metal Specificity of Metallosensors. In *Encyclopedia of Inorganic and Bioinorganic Chemistry*; John Wiley and Sons, Ltd.: Chichester, UK, 2013; pp. 209–224. Copyright (2013) John Wiley and Sons).

Several crystal structures of NikR proteins from *E. coli* [45–48], *Pyrococcus horikoshii* (*P. horikoshii*) [49], and *H. pylori* [50–54] show that the protein is a homotetramer. The protein has two ribbon-helix-helix DNA binding domains (DBDs) attached by a flexible linker at either end of the tetrameric C-terminal metal-binding domain (MBD) [45]. NikR binds one Ni(II) per monomer in the MBD and is the sole member of the ribbon-helix-helix (β-α-α) family of prokaryotic DNA-binding proteins that is regulated by a metal [55]. The crystal structures of NikR homologues show NikR in different conformations (open, trans, and cis) depending on the orientation of the DBDs with respect to the MBD (Figure 3). An open conformation where the DBDs are linearly placed in each side of the MBD was observed for the apo structures from *E. coli* (Figure 3A) and *P. horikoshii* [45,49]. The nickel-bound *P. horikoshii* and *H. pylori* (Figure 3B) structures feature a closed trans-conformation, where the DBDs are on opposite sides of the MBD [49,54]. The *P. horikoshii* NikR protein was crystallized from a solution containing the apo protein and nickel chloride, while the *H. pylori* NikR protein was obtained by soaking apo *H. pylori* NikR crystals with nickel sulfate [49,54]. The *E. coli* nickel- and DNA-bound NikR structure, obtained from Ni(II)-NikR using the hanging drop vapor diffusion method in a drop consisting of 2 μL of NikR–DNA complex, reveals a closed cis conformation with the DNA-binding domains on the same side of the C-terminal metal binding domain (Figure 3C) [46]. It is still unclear if these conformational changes exist in solution. Nuclear magnetic resonance (NMR) experiments coupled with molecular dynamic simulations suggest that NikR is capable of interconverting between the open, trans, and cis conformations and that these interconversions are facilitated by Ni(II) [56]. However, Small-angle X-ray Scattering (SAXS) experiments done on *H. pylori* NikR do not support these large conformational changes [50].

The *E. coli* NikR DNA bound crystal structure shows that there are two distinct metal-binding sites, four “high affinity” metal binding sites located in the MBD and two “low affinity” metal-binding sites located near the interface of the C-terminus and the DBD (Figure 3C) [46]. Nickel ions bind to the high affinity sites in a four-coordinate planar geometry [46,57] (Figure 3D). The nickel ion is bound by the side chains of His87, His89, and Cys95 from one NikR monomer, and by His76 from an adjacent monomer (Figure 3D) [46]. The structure of *E. coli* Ni(II) NikR bound to DNA shows that potassium ions are coordinated in a bidentate manner by Glu30 and Asp34, and by the backbone carbonyl oxygens of Ile116, Gln118, and Val121 (Figure 3D) [46]. XAS studies reveal that Ni(II) ions bind to the “low affinity site” site with octahedral geometry [57]. Like, *E. coli* NikR, *H. pylori* NikR also binds four nickel ions in the high-affinity sites with four-coordinate planar geometry [51]. Isothermal titration calorimetry (ITC) studies determined that the low-affinity sites in *H. pylori* NikR can bind up to ten nickel ions [58].

The binding affinities of Ni(II) ion to the “high-affinity site” of NikR have been determined in both *E. coli* at pH 7.5 and 7.6, and *H. pylori* at pHs 6.5, 7.0, 7.5 and 8.0 by competition studies and ITC, respectively [38,58,59]. A K_d_ of 10^−12^ M was determined for *E. coli* NikR, while *H. pylori* NikR had dissociation constants of 10^−9^ M [38,58,59]. The differences in affinities determined for NikR may be attributed to the differences in the experimental conditions and the techniques as *E. coli* NikR ITC experiments that used conditions identical to those used for *H. pylori* NikR determined a dissociation constant of 10^−9^ M [37]. A K_d_ of 29 X 10^−9^ M was determined for the “low-affinity site” by titrating NikR loaded with 4 Ni(II)/monomer with excess Ni(II) ions [39]. A similar K_d_ was determined using ITC but features 4 Ni(II) binding to the “low-affinity” site [37].

There is no thermodynamic metal preference for Ni(II) ions binding to *E. coli* NikR as it binds to a variety of transition metal ions in vitro in the “high-affinity” site, and the binding affinities follow the Irving Williams series (Co(II) < Ni(II) < Cu(II) > Zn(II) [47,57,59]. Nonetheless, in vivo, the protein only responds to the binding of Ni(II) ions. Ni(II) loaded NikR is less susceptible to chemical and thermal denaturation than Mn(II)-, Co(II)-, Cd(II)-, Cu(II)- or Zn(II)-NikR [59]. X-ray absorption spectroscopy (XAS) studies done on NikR showed that the non-cognate metals, Co(II), Cu(I), Cu(II), and Zn(II) adopt six-coordinate octahedral, three-coordinate trigonal, four-coordinate planar, and four-coordinate tetrahedral geometries, respectively [57]. Similar to Ni(II), the Cu(II) site is also four-coordinate planar but has a slightly different ligand set [57]. Based on the XAS studies it can be concluded that in *E. coli* NikR metal ion selectivity is achieved by the coordination number and geometry of the metal protein complex as well as ligand selection [57].

### 2.2. Nur

**Figure 4.**
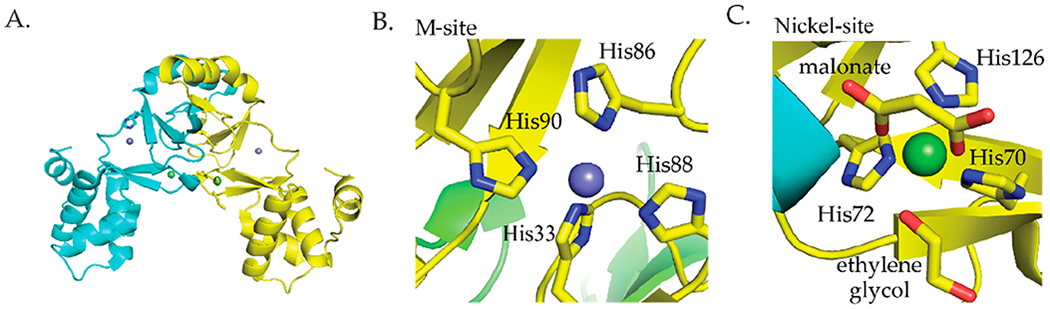
(**A**) The crystal structure of *S. coelicolor* Nur (PDB ID 3EYY) [62]. The monomers are colored cyan and yellow. Zinc ions and nickel ions are shown as slate spheres and green spheres, respectively. (**B**) Close-up of the M-site. (**C**) Close-up of the Ni-site. (Figure adapted with permission from Higgins, K.A.; Carr, C.E.; Maroney, M.J. Specific Metal Recognition in Nickel Trafficking. *Biochemistry* **2012**, *51*, 7816–7832. Copyright (2012) American Chemical Society.).

*Streptomyces coelicolor* (*S. coelicolor*) Nur is a member of the Fur family of metalloregulators that controls nickel homeostasis and oxidative stress response [60]. Nur responds to the binding of Ni(II) and represses the *sodF* gene that encodes an iron-containing superoxide dismutase (FeSOD) and the *nikABCDE* gene cluster encoding components of a nickel transporter [60,61]. Additionally, Nur induces the transcription of *sodN* that encodes a nickel-containing superoxide dismutase (NiSOD) [60,61]. Nur was crystallized from solutions containing the apo protein with zinc chloride or nickel chloride. The crystal structure shows that Nur is a homodimeric protein with two metal binding sites per monomer [62] (Figure 4A). The first site, the M site, binds nickel ions using four histidine ligands in a four-coordinate square planar geometry (Figure 4B) [62]. The second site, the nickel site, coordinates Ni(II) with octahedral geometry using three histidine ligands, two oxygen ligands from malonate, and one oxygen ligand from ethylene glycol (Figure 4C) [62]. It is important to note that this site may be an artifact of crystallization conditions and may not reflect the actual coordination environment of Ni(II) binding to this site of Nur [62]. ITC studies determined that 4 Ni(II) ions bind per dimer with two binding events that have dissociation constants of 10 nM and 280 nM, respectively [37].

### 2.3. RcnR/Csor Family

**Figure 5.**
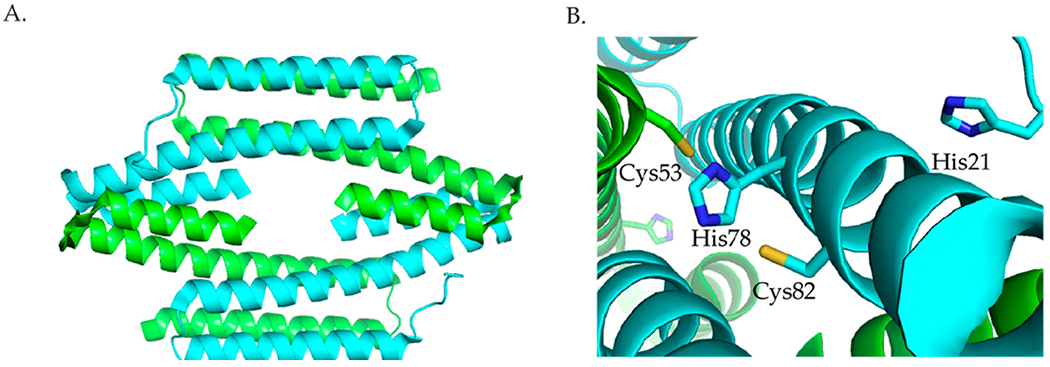
(**A**) The crystal structure of *Synechocystis* PCC6803 InrS (PDB ID 5FMN) [64]. The monomers in each dimer are colored in blue and green. (**B**) Highlights the metal binding pocket of InrS and important residues for Ni(II) binding to the protein. (Figure adapted with permission from Springer Nature Customer Service Centre GmbH: Springer Nature, Nature Chemical Biology. Foster, A.W.; Pernil, R.; Patterson, C.J.; Scott, A.J.P.; Palsson, L.O.; Pal, R.; Cummins, I.; Chivers, P.T.; Pohl, E.; Robinson, N.J. A tight tunable range for Ni(II) sensing and buffering in cells. *Nat. Chem. Biol.* **2017**, *13*, 409–414. Copyright (2017)).

**Figure 6.**
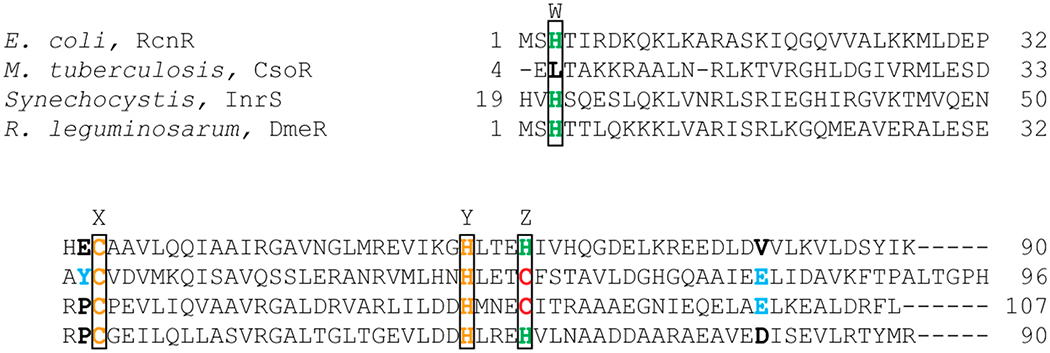
Sequence alignment of *E. coli* RcnR with *M. tuberculosis* CsoR, *Synechocystis* PCC 6803 InrS, and *R. leguminosarum* DmeR. Fingerprint residues (W,X,Y,Z) found in all four proteins are shown in orange while those that are unique to CsoR and RcnR are in red and green, respectively. The residues that form a hydrogen bonding network with His61, Tyr35 and Glu81, in *M. tuberculosis* CsoR are shown in blue, The sequence alignment was generated with Clustal Omega [71].

Three members of the RcnR/CsoR family of metalloregulators are nickel responsive. The crystal structures of the Cu(I) bound *Mycobacterium tuberculosis* (*M. tuberculosis*) CsoR, a Cu(I)-responsive metalloregulator, and *Synechocystis* sp. PCC68 InrS (Figure 5) with no metal bound show that proteins in this family are an all α-helical dimer of dimers [63,64]. In *M. tuberculosis,* Cu(I) is bound in a trigonal geometry by Cys36 from one subunit, and His61 and Cys65 from another subunit [63]. Sequence alignment (Figure 6) shows that these residues are the same in InrS, but in RcnR, and DmeR Cys65 of CsoR corresponds to a His. The CsoR crystal structure showed that there was a hydrogen bonding network between His61 and Glu81 from one subunit and Tyr35 from another subunit [63]. This hydrogen bonding network was shown to be important for allosterically coupling Cu(I) binding to DNA binding [63,65].

#### 2.3.1. RcnR

*E. coli* RcnR is a Ni(II)- and Co(II)-responsive metalloregulator that regulates the expression of the Ni(II) and Co(II) exporter and periplasmic protein RcnAB [7,8,66]. Apo-RcnR binds to a TACT-G_6_-N-AGTA sequence of which two are located in the *rcnA-rcnR* intergenic region [67,68]. Computational models suggest that RcnR is composed of three α-helices in each monomer; these monomers come together to form a dimer, and the tetrameric oligomer is formed by the dimerization of two dimers [37]. RcnR binds one Ni(II) or Co(II) ion per monomer with nanomolar affinity [69]. Similar to NikR, ITC studies determined that the 4 Ni(II) ions bind to the protein tetramer in two binding events [37]. The protein binds the cognate metals, Ni(II) and Co(II), with six-coordinate octahedral geometry and noncognate metals, Cu(I) and Zn(II), with three- and four-coordinate geometry [69,70]. Likewise, NikR metal ion selectivity is achieved by the coordination number and geometry of the metal protein complex.

Mutagenesis studies coupled with XAS studies suggest that the N-terminal amine, His3, Glu34, Cys35, Glu63, and His64 bind to Co(II) forming a CoSNH_2_(N_Im_)_2_(O)_2_ site, this arrangement of the cobalt site is supported by computational models [70,72,73]. Similarly, the Ni(II) site is composed of the N-terminal amine, Cys35, Glu63, His64, and two other N/O ligands, one of which may be Glu34,forming a NiSNH_2_ON_Im_(N/O)_2_ site [70,72,73]. Computational models show that most of the metal binding residues, the N-terminal amine, His3, Glu63, His64, are derived from one monomer, while Glu34 and Cys35 are derived from another monomer [73]. The Ni(II) and Co(II) sites also differ in M-S bond distances, the number of histidine ligands, and the number of Glu ligands. XAS studies determined that the M-S bond distance is longer for Ni(II) compared to Co(II), 2.62(3) Å versus 2.31(2) Å, respectively [70].

Mass spectrometry, NMR, metal binding studies, and modeling studies suggest that cognate metal binding remodels the shape of the tetramer in a way that does not allow for the relevant positively charged residues to interact with the DNA [36,74,75]. Hydrogen-deuterium exchange studies coupled with mass spectrometry (HDX-MS) was used to probe the RcnR structure in the presence of DNA, Ni(II), Co(II), or Zn(II) [76]. These experiments determined that Ni(II) and Co(II) binding to RcnR orders the N-terminus, decreases the flexibility of helix 1, and induces conformational changes in the protein that restricts DNA interactions with Arg14 and Lys17 [76].

#### 2.3.2. InrS

The second Ni(II) metalloregulator, InrS, belonging to the RcnR/CsoR family was identified in *Synechocystis* sp. PCC6803 [77]. InrS regulates the transcription of the genes encoding the Ni(II) efflux protein NrsD [77]. *Synechocystis* lacks the *nikR* gene, however, InrS also plays a role in Ni(II) import as it binds upstream of the *nik* operon and enhances the expression of the genes encoding nickel-import machinery [64].

InrS binds one stoichiometric equivalent of Ni(II) per monomer [77]. Surprisingly, InrS possesses all the residues known to bind Cu(I) in *M. tuberculosis* CsoR, Cys53, Cys82, and His78 (equivalent to Cys36, Cys65 and His61 in *M. tuberculosis* CsoR) (Figures 5 and 6) but InrS is responsive to Ni(II) [63,77]. It was determined by mutagenesis studies that these residues are important for Ni(II) binding to InrS [64,78]. The crystal structure (Figure 5) shows that these residues are located in the α2 helix [64]. His21 (analogous to His3 in RcnR) (Figure 6) is located on the flexible N-terminal extension and is capable of approaching the Ni(II)-binding site [64]. XAS spectroscopy determined that the nickel site is four-coordinate planar and that His21 is a Ni(II) ligand in InrS [79]. The protein binds Ni(II) using His21, Cys53, His78, and Cys82 forming a Ni(N_Im_)_2_(S)_2_ complex [79]. The protein can also bind 1 Cu(I) per monomer or 1–1.5 Co(II) per monomer [77]. The metal site structure of InrS are different from those observed for RcnR and are similar to those of CsoR as they feature a square planar geometry for Ni(II) and tetrahedral geometry for Co(II) [63,70,77,80].

Competition experiments with Ni(II) chelators EDTA, NTA, and EGTA determined that InrS binds Ni(II) tighter than RncR with an affinity of 10^−14^ M [77]. However, dissociation constants obtained from ITC determined that the binding affinities of InrS to Ni(II) are similar to those determined for RcnR with values of 70 × 10^−9^ M and 4.5 × 10^−6^ M [37]. Like NikR and RcnR, InrS binds 4 Ni(II) in two binding events. InrS also binds Cu(I) and Zn(II) tightly with affinities of 10^−18^ and 10^−13^ M, respectively [78]. Like CsoR, InrS also has a glutamate residue, Glu98, that aligns with Glu81 of CsoR, which was shown to be important for the hydrogen bonding network that links Cu(I) binding to DNA binding [63,65]. Work done by Foster et al. showed that an E98A mutant InrS protein binds Ni(II) like the wild-type protein and binds DNA tighter than the wild-type protein in the presence of Ni(II) [78]. This result suggests that, unlike CsoR, Glu98 is not essential for allostery in InrS.

#### 2.3.3. DmeR

DmeR is a Ni(II)- and Co(II)-responsive metalloregulator that has been identified in *Rhizobium leguminosarum (R. leguminosarum), Agrobacterium tumefaciens* (*A. tumefaciens*), *Sinorhizobium meliloti* [81–83]. The *dmeRF* operon encodes both DmeR and DmeF, a cation diffusion facilitator (CDF) [81]. DmeR binds to an AT-rich inverted repeats sequence. DmeR has a RcnR-like finger print, HCHH (Figure 6), and is therefore, expected to bind Ni(II) and Co(II) with six-coordinate octahedral geometry as seen in RcnR. It is interesting to note that cobalt stress induces the expression of iron-responsive genes in *A. tumefaciens* [82]. Additionally, RirA, an iron regulator, is essential for the bacteria to cope with nickel and cobalt toxicity [82].

### 2.4. NmtR

**Figure 7.**
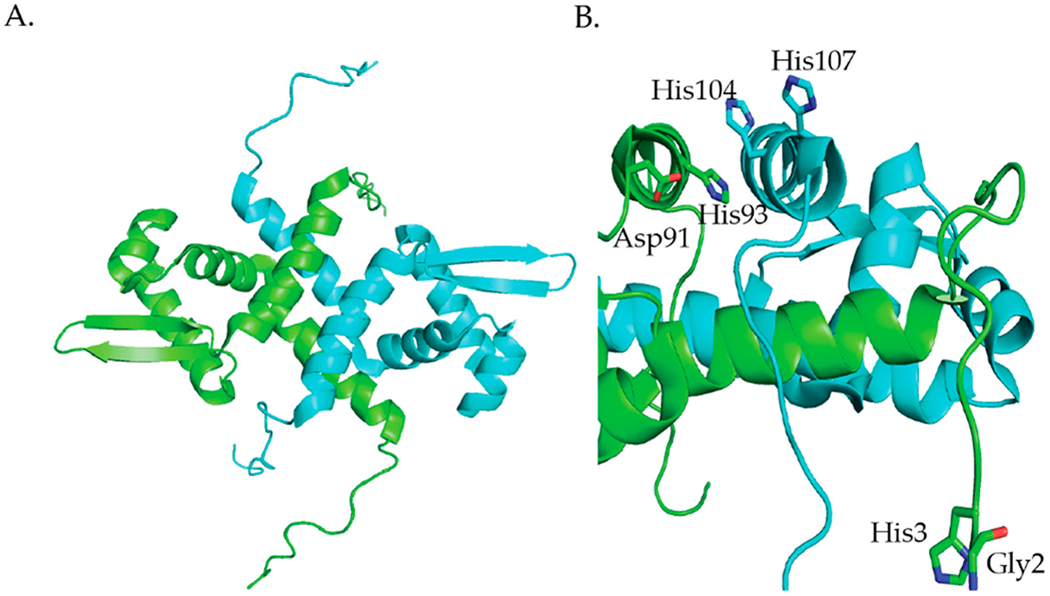
(**A**) NMR structure of apo *M. tuberculosis* NmtR (PDB ID 2LKP) [87]. NmtR monomers are colored in green and blue (**B**). Close-up of one of the nickel sites showing the nickel binding site. (Figure adapted with permission from Lee, C.W.; Chakravorty, D.K.; Chang, F-M.J.; Reyes-Caballero, H.; Ye, Y.; Merz, K.M.; Giedroc, D.P. Solution structure of *Mycobacterium tuberculosis* NmtR in the apo state: Insights into Ni(II)-mediated allostery. *Biochemistry* **2012**, *51*, 2619–2629. Copyright (2012) American Chemical Society).

*M. tuberculosis* NmtR is a Ni(II)- and Co(II)-responsive transcriptional metalloregulator that belongs to the ArsR/SmtB family [84]. NmtR controls the expression of the *nmt* operon that encodes a P-type ATPase metal efflux pump, NmtA [85]. NmtR binds 2 Ni(II) per dimer with K_ds_ of 8.7 × 10^−11^ M and 1.4 × 10^−10^ M [86]. The NMR structure of apo-KmtR (Figure 7) shows the winged-helix fold (α1-α2-α3-αR-β1-β2-α5) that is typical of the ArsR/SmtB family of transcriptional metalloregulators [87]. Additionally, NmtR has long N-terminal and C-terminal extensions (Figure 7) [87]. Like RcnR, NmtR binds its cognate metals with higher coordination numbers than noncognate metals. NmtR binds Ni(II) with six-coordinate geometry and Co(II) with five- or six-coordinate geometry [85]. Additionally, Zn(II) binds to NmtR with four-coordinate geometry [85]. NmtR is the third example in this review of a metalloregulator that achieves metal ion selectivity through the coordination number and geometry of the metal site structure. A combination of molecular dynamics simulations, quantum chemical calculations, metal binding studies and mutagenesis studies confirms that the Ni(II) binds to NmtR with six-coordinate geometry binding to the N-terminal amine, and the side chains of His3, Asp91, His93, His104, and His107 (Figure 7B) [86,87].

### 2.5. Other Transcriptional Metalloregulators

Nickel responsive transcriptional metalloregulators have been identified in other bacteria but the metal site structures have not been characterized. These include: SrnRQ, KmtR, NcrB, Mua, and NimR. In *Streptomyces griseus*, the expression of *sodF*, which encodes an iron- and zinc-containing superoxide dismutase is regulated by two nickel responsive regulators SrnR and SrnQ that work together [88]. SrnR is a member of the ArsR/SmtB family while SrnQ does not show any similarities to any known proteins [88]. SrnR and SrnQ form a complex both in the presence and absence of nickel with maximum interaction shown when they are in a 1:1 stoichiometric ratio [88]. The two proteins come together to form an octamer that is composed of four subunits from each protein [88]. A second Ni(II)- and Co(II)-responsive transcriptional regulator, KmtR, was identified in *M. tuberculosis* [89]. Apo KmtR represses the expression of a putative CDF metal exporter [89]. *Leptospirillum ferriphilum* UBK03 NcrB is a histidine rich protein that regulates the expression of genes associated with nickel export *ncrAC* [90]. Mua binds nickel and modulates urease activity in *H. pylori* [91]. The protein binds two nickel per dimer and represses urease activity at high concentrations [91]. *Haemophilus influenzae* NimR is a regulator belonging to the MerR family that binds one Ni(II) ion per dimer [92]. NimR regulates the expression of a Ni(II) uptake transporter, NikLMQO [92].

## 3. Ni(II) Chaperones Associated with [NiFe]-Hydrogenase and Urease

The nickel chaperones HypA, HypB, SlyD, and UreE have been characterized individually and in complexes from various organisms including *E. coli* and *H. pylori*. Several studies have shown that these proteins interact with each other to deliver nickel to [NiFe]-hydrogenase. These studies are discussed in this section. Table S2 lists the respective K_d_s and the pH at which the K_d_ was determined, the coordination number (CN #), and the ligands used to coordinate the metal ion in HypA, HypB, SlyD, and UreE.

### 3.1. HypA

**Figure 8.**
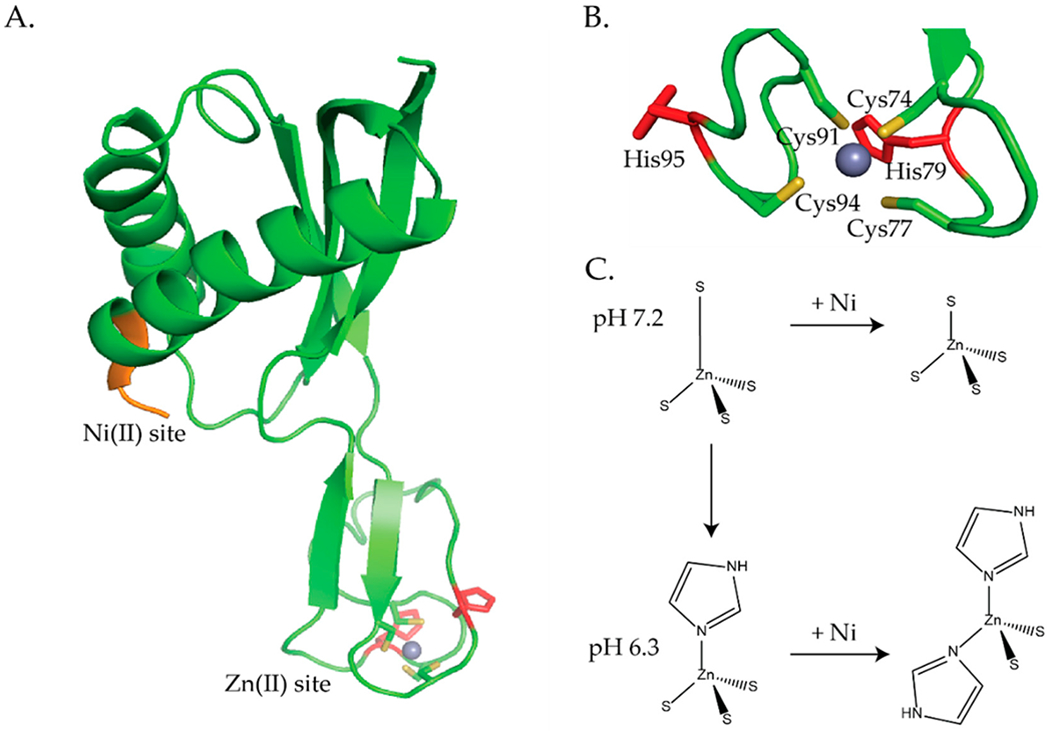
NMR structure of *H. pylori* HypA at pH 7.2 (PDB ID 6G81) [100]. HypA was expressed in growth medium containing zinc sulphate and purified as ZnHypA [100]. (**A**) The nickel binding MHE residues are in orange and the zinc site is shown at the base of the protein where the zinc ion is coordinated by 4S-donors forming a tetrahedral zinc site. The histidine residues flanking the CXXCX*_n_*CXXC motif are shown in red. (**B**) Close-up of the zinc site. (**C**) Scheme depicting the changes in the zinc site when the pH changes and nickel binds to HypA (This scheme is adapted with permission from Herbst, R.W.; Perovic, I.; Martin-Diaconescu, V.; O’Brien, K.; Chivers, P.T.; Pochapsky, S.S.; Pochapsky, T.C.; Maroney, M.J. Communication between the zinc and nickel sites in dimeric HypA: metal recognition and pH sensing. *J. Am. Chem. Soc.* **2010**, *132*, 10338–10351. Copyright (2010) American Chemical Society).

**Figure 9.**
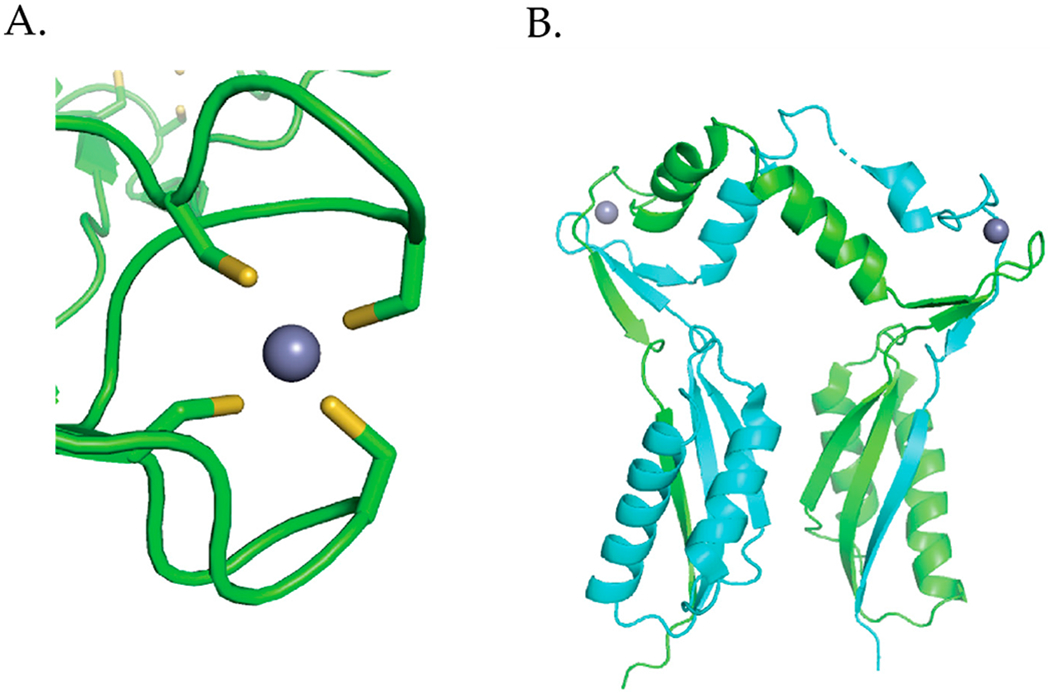
Crystal structure of HypA from *T. kodakarensis* [99]. The protein monomers are shown in cyan and green and the zinc ion is shown as a slate sphere. (**A**) The zinc site from the monomeric protein (PDB ID 3A43) [99]. (**B**) Crystal structure of the domain swapped dimer (PDB ID 3A44) [99]. (Figure adapted with permission from Higgins, K.A.; Carr, C.E.; Maroney, M.J. Specific Metal Recognition in Nickel Trafficking. *Biochemistry* 2012, *51*, 7816–7832. Copyright (2012) American Chemical Society).

HypA is a nickel chaperone that is involved in delivering nickel to [NiFe]-hydrogenase and in the case of *H. pylori* it is involved in nickel delivery to urease [32]. Homologous HypA proteins have been characterized from *E. coli, H. pylori*, and *Thermococcus kodakarensis (T. kodakarensis). E. coli* HypA and its homologous protein, HybF are essential for the maturation of [NiFe]-hydrogenases [18,93]. HypA proteins have been reported as being a dimer in solution based on elution volumes [94,95], however, size exclusion chromatography in combination with multiple light scattering (SEC-MALS) determined that the protein is a monomer in solution [96]. HypA proteins feature two metal binding sites, a N-terminal nickel site and a zinc site [94,95,97–100]. The HypA structures reveal that the nickel binding site and the zinc binding site are separated by a long flexible linker (Figures 8 and 9) [98–100]. The N-terminal nickel site features a highly conserved MHE motif, which includes the first three amino acids at the N-terminus of the protein, that is used to bind two Ni(II) ions per dimer with micromolar affinity [93–96,98,101]. A K_d_ of 75 ± 46 nM was reported by Douglas et al. for a Strep-tagged HypA protein binding to nickel in competition with a well-established metal binding indicator, Mag-fura 2 [102]. Mutagenesis studies showed that His2 located in the MHE motif is critical for nickel binding, [NiFe]-hydrogenase activity, and, in the case of *H. pylori*, it is important for urease activity [95]. HypA utilizes two conserved CXXC motifs to bind one zinc ion per monomer [93,94,97–101] with nanomolar affinity [94].

*H. pylori* HypA binds Ni(II) with a K_d_ of ~1 μM at pH 7.2 and this binding is weaker at pH 6.3 [96,97,103]. There are some discrepancies regarding the coordination number and geometry of the nickel biding site determined by XAS and X-ray crystallography. XAS studies determined that the Ni(II) site is six-coordinate and Ni(II) is ligated by 6 N/O-donors of which 1–2 are histidines [97,104]. The first NMR structure of the monomeric protein from *H. pylori* features an N-terminus that is modified by two additional residues, Gly and Ser, that were left over after thrombin cleavage of a histidine tag [98]. UV-vis studies done on this protein determined that nickel binds to *H. pylori* HypA in a planar coordination with 4N/O-donors. Initial NMR studies revealed that the Ni(II) binding site is located at the N-terminus and that nickel is coordinated by the backbone nitrogen of His2, Glu3, and Asp40 as well as the imidazole from His2 [98]. Additional NMR studies on the unmodified HypA protein revealed nickel binding to HypA resulted in dipolar broadening in the N-terminal region. This result is consistent with a paramagnetic (*S* = 1) five- or six-coordinate nickel site [97]. The latter six-coordinate Ni(II) site is supported by density functional theory (DFT) calculations, which suggest that Ni(II) is bound by the N-terminal amine, the amide nitrogen of His2 and Glu3, and the sidechains of Glu3, Asp40, and His2, forming a NiNH_2_(N)_2_O_2_Ni_Im_ site [100]. XAS studies done by Hu et al. on the unmodified protein confirmed that the nickel site is six-coordinate and that the nickel ion is coordinated by 2N/O-donors, 1 imidazole, the N-terminal amine, and 2 backbone amide [96]. Additionally, the binding of the N-terminal amine to nickel was confirmed with a L2* HypA mutant protein. This mutation features the insertion of Leu into position two so that the MHE motif becomes MLHE [96]. The L2*HypA protein changes the coordination environment of the Ni(II) ion, the Ni(II) site is five-coordinate Ni(II) site with a Br^−^ ligand from the buffer [96]. The presence of the Br^−^ ligand bound to Ni(II) suggests that the MLHE mutant HypA protein results in an open coordination position on Ni(II), which is occupied by an anion from the buffer.

The zinc site consists of a loop with a CXXCX*_n_*CXXC motif that coordinates Zn(II) ions in a four-coordinate tetrahedral geometry (Figure 8A,B and Figure 9A) [93,94,97–101]. The *H. pylori* NMR structures show that the Zn(II) ion is coordinated by 4 S-donors from Cys74, Cys77, Cys91, and Cys94 (Figure 8A,B) [98,100]. The crystal structure of HypA from *T. kodakarensis* was solved in both the monomeric and dimeric forms (Figure 9) [99]. The zinc site in the monomeric form confirms that zinc is coordinated by the CXXCX*_n_*CXXC motif (Figure 9A), however, the structure of the dimeric protein shows that there is a domain swap in the homodimeric protein that affects the cysteine coordination of the zinc site (Figure 9B) [99]. The physiological role of this domain swap is unknown [99]. In the dimeric form the zinc is coordinated by only 1 CXXC motif from each monomer [99]. Additionally, the crystallographic studies revealed that the HypA zinc site is dynamic [99].

XAS studies also revealed that the zinc site is dynamic and senses both pH changes and nickel binding to the protein (Figure 8C) [97]. In agreement with the NMR structures, the XAS studies determined that at pH 7.2, the zinc site is four-coordinate, tetrahedral, with 4 S-donors, (Zn(S)_4_) (Figure 8B,C) [97,98,100]. Similarly, at pH 6.3 (~the internal pH of *H. pylori* under acid shock), the zinc site is also four-coordinate but features 3 S-donors and 1 imidazole ligand from a histidine (Zn(S)_3_(N_Im_)) (Figure 8C) [97]. ITC studies determined that this structural change altered the nickel binding stoichiometry and binding affinity. At pH 6.3, ZnHypA binds 1 Ni(II) ion per dimer with a K_d_ of 17 μM versus 1 Ni(II) ion per monomer at pH 7.2 with a K_d_ of 1 μM [97]. When ZnHypA binds Ni(II) at pH 7.2 the structural changes in the zinc site are subtle, the zinc site is still four-coordinate, but 1 of the Zn-S bonds is shortened (Figure 8C) [97]. At pH 6.3, the changes in the zinc site are more pronounced when Ni(II) binds to ZnHypA; 2 of the S-donor ligands at the zinc site are replaced by 2 imidazole ligands from histidines forming a (Zn(S)_2_(N_Im_)_2_ structure (Figure 8C) [97]. In *H. pylori* the two CXXC motifs are flanked by two histidine residues, His79 and His95 (Figure 8A,B). Mutating any of the Cys residues in CXXC or His79 or His95 resulted in a static zinc site that no longer senses pH changes or Ni(II) binding to HypA [97]. Cys to Ala or Asp mutations resulted in a zinc site that was four-coordinate with 2 S-donors and 2 imidazoles ((Zn(S)_2_(N_Im_)_2_) [97]. This zinc site is indistinguishable from the ZnHypA site with Ni(II) bound at pH 6.3 (Figure 8C) [97]. Alanine substitutions for His79 and His95 also resulted in a zinc site that featured 4 S-donor ligands, (Zn(S)_4_), as seen for the wild-type HypA zinc site in the presence of Ni(II) at pH 7.2, which suggests that His79 and His95 are the residues that bind the wild-type NiZnHypA protein at pH 6.3 (Figure 8C) [97].

In vivo studies done on *H. pylori* mutant strains determined that the dynamic zinc site in HypA is important for the acid viability of the bacterium, urease activity, and [NiFe]-hydrogenase activity [33,105]. However, the conformational changes in the zinc site observed by XAS studies when Ni(II) binds to HypA are not supported by NMR studies [100]. *H. pylori* mutant strains were created that involved mutating individual residues in the CXXC motifs, Cys74, Cys77, Cys91, and Cys94 to Asp and Ala, as well as His79 and His95 to Ala [33]. *H. pylori* strains of these 10 *hypA* mutants, a *hypA* mutant (*hypA::kan-sacB*), a *hypA*-restorant (hypA-R) used as a control for any defects that may have resulted from genetic manipulation, and a ureB mutant ΔureB, used as a control) all survived when resuspended in phosphate buffered saline (PBS) at pH 6 both in the absence and presence of urea [33]. However, in PBS buffer at pH 2.3 a significant decrease in acid resistance was observed for C74D, C91A, and C94A *hypA* mutant strains compared to the wild type strain [33]. All the *H. pylori* mutant strains involving the Cys residues resulted in a decrease in urease activity (15% activity compared with the *hypA*-restorant strain) [33,97]. The C77D, C91D, and C94D mutant strains had moderate effects on urease activity (12–13% compared with the hypA-restorant strain), while C74A, C74D, C77A, C91A, and C94A had severe effects on urease activity (similar to the ΔhypA strain and >4% compared to the hypA-restorant strains) [33]. The His mutations had no effect on urease activity [33]. Johnson et al. suggest that the cysteine residues play a critical role in acid viability and mutating these residues affects the ability of HypA to supply the urease maturation pathway with nickel [33]. Studies done by Blum et al. determined that [NiFe]-hydrogenase activity does not contribute to acid resistance in *H. pylori. H. pylori* mutant strains similar to the ones used above in the acid viability and urease study were used to measure [NiFe]-hydrogenase activity [105]. The C74A, C74D, C91A, and C94A mutations all resulted in a severe decrease (9–15% of wild type activity) in [NiFe]-hydrogenase activity, whereas, only a moderate decrease (36–60% of wild type activity) in [NiFe]-hydrogenase activity was observed for C77A, C77D, C91D, and C94D mutant strains [105]. The H79A and H95A mutant strains had little effect on [NiFe]-hydrogenase activity, 90% and 110% of wild type activity, respectively [105]. These results coupled with the XAS studies [97,104] suggest a regulatory role for the zinc site [33,97,104,105] and it has been suggested that changes in the zinc site may affect interactions between HypA and other nickel proteins [33,97] and the delivery of nickel to [NiFe]-hydrogenase and urease [33,105]. This dynamic zinc site appears to be unique to *H. pylori* HypA as other HypA homologues, including *T. kodakarensis* HypA, do not have conserved histidine residues flanking the CXXCX*_n_*CXXC motif [17,101]. Additionally, the crystal structures of *T. kodakarensis* HypA show that there is no change in the structure of the zinc binding domain when Ni(II) binds to the protein [101].

Nickel binding to the MHE motif is also important for both [NiFe]-hydrogenase and urease activity [33,95,105]. A H2A *hypA* mutant strain of *H. pylori* was created by replacing the His codon with the codon for Ala. [95]. This mutant strain of *H. pylori* lacked [NiFe]-hydrogenase activity and only had 2% of the wild-type urease activity [95]. NMR studies showed that the H2A mutant protein had a structure that was similar to the wild-type protein but nickel binding experiments determined that this mutant protein did not bind nickel [95]. Additionally, the L2* *hypA* mutant strain of *H. pylori* is sensitive to acid and has decreased urease activity [96]. Furthermore, in *H. pylori hypA*, the L2* mutation resulted in a decrease (14% of wild type activity) in [NiFe]-hydrogenase activity [105].

HypA has been shown to form complexes with HypB in *E. coli, H. pylori*, and *T. kodakarensis* [94,95,101,106]. Three crystal structures have been solved of the HypAB complex from *T. kodakarensis* (vide infra). One structure is in the presence of adenosine 5′-*O*-(3-thiotriphosphate) (ATP;S) and nickel ions, the other with beta,gamma-methyleneadenosine-5′triphosphate (AMPPCP) (a nonhydrolyzed ATP analog) and Ni(II) ions, and the third with just AMPPCP [101]. The overall architecture of the proteins is similar with small differences in the local conformations. Additionally, there are crystal structures of HypA complexed with the [NiFe]-hydrogenase large subunit in an immature state, HyhL, from *T. kodakarensis* [107]. The HyhL-HypA complex was prepared by mixing HyhL and HypA in a 1:1.4 molar ratio [107]. The proteins were incubated for 6 days at 20 °C, followed by further incubation for 1 day at 4 °C [107]. Analysis of the HyhL-HypA structure revealed that the nickel metal binding sites of the two proteins are in close proximity and that the N-terminal tail of HyhL interacts with the nickel binding site on HypA [107].

### 3.2. HypB

HypB is a member of the G3E family of P-loop GTPases [108]. All HypB proteins have a conserved GTPase domain (G-domain), which contains a low affinity metal binding site than can bind either nickel or zinc [109–114]. The GTPase activity of HypB is required for nickel insertion and maturation of [NiFe]-hydrogenase and plays a role in urease maturation [95,115,116]. In *E. coli*, GTPase hydrolysis controls nickel but not zinc binding to HypB and protein–protein interactions between HypA and HypB [117]. *E. coli* HypB binds two metal ions per monomer in two very distinct metal sites, both of which are critical for [NiFe]-hydrogenase maturation [109,110]. The high affinity site, which is unique to *E. coli* HypB, binds Ni(II) with a K_d_ in the subpicomolar range, is located at the N-terminus of the protein, and features a CXXCGC motif [109]. The second site, a low affinity site, located in the GTPase domain, has a higher affinity for Zn(II) (K_d_ 1 μM vs 12 μM for Ni(II)) [109]. The GTPase activity of *E. coli* HypB is low, with a k_cat_ 0.17 min^−1^ and a k_m_ of 4 μM [116].

XAS studies determined that Ni(II) binds to the high affinity site and low affinity site in a four-coordinate planar geometry and six-coordinate octahedral geometry, respectively [110]. Nickel binds to the high affinity site utilizing the 3 S-donors from the 3 N-terminal cysteines, Cys2, Cys5, and Cys7, as well as 1 N/O donor from the N-terminal amine to form a Ni(S)_3_NH_2_ structure [109,110,118]. Nickel binds to the low affinity site using Cys166, His167, and 4 other N/O-donating residues [109,110]. XAS studies revealed that metal binding to the high affinity site results in structural changes at the low affinity site [110]. Likewise, metal binding to the low affinity site results in structural changes at the high affinity site [110]. X-ray absorption near-edge spectroscopy (XANES) analysis shows that when Zn(II) binds to the low affinity site, a less intense 1s → 4p_z_ transition is observed, and the peak at 8342 eV is slightly more intense [110]. This indicates that there is a slight distortion in the planar geometry and a reduction in the S-donor ligand content of the high affinity site [110]. Extended X-ray absorption fine structure (EXAFS) analysis of Ni(II) in the high affinity site in the absence of Zn(II) bound to the low affinity site shows the Ni(II) coordinated by 3 S-donors at a distance of 2.17 Å and 1 N/O-donor at 1.87 Å, forming a Ni(S)_3_(N/O) structure [110]. When zinc binds to the low affinity site these bond distances are altered, the Ni-S bond distance is slightly shorter at 2.15 Å and the Ni-N/O bond distance is longer at 2.02 Å [110]. A Ni(II) site with one less S-donor, Ni(S)_2_(N/O)_2_, that is in agreement with the XANES analysis of a decrease in the S-donor ligand content involving 2 S-donors at 2.17 Å and 2 N/O-donors at 2.02 Å, was also obtained for Ni(II)Zn(II) HypB but this fit had a similar F-factor (a goodness of fit parameter) to the 3S 1N/O fit [110]. Based on the EXAFS data, it is not clear if a S-donor is replaced by a N/O-donor when Zn(II) bind to the low affinity site. Similarly, changes in the low affinity site, the G-domain, were observed when Ni(II) binds to the high affinity site. XANES analysis showed that the geometry of the zinc site is not altered when Ni(II) binds as it is four-coordinate tetrahedral regardless of Ni(II) being present in the high affinity site [110]. When Ni(II) binds to the high affinity site the coordination environment of Zn(II) in the low affinity site changes from Zn(S)_2.5_N/OZn(N_Im_)_0.5_ to Zn(S)_2_N/OZn(N_Im_). EXAFS analysis revealed that in the absence of Ni(II), the zinc site contains 2 Zn(II) ions with an average of 2.5 S-donors at 2.29 Å, 1 N/O-donor at 2.02 Å, 0.5 imidazole nitrogen ligand at 1.98 Å, and a Zn-Zn interaction at 4.01 Å [110]. When Ni(II) is bound to the high affinity site, the zinc site still contains 2 Zn(II) ions with an average of 2 S-donors at 2.29 Å, 1 N/O-donor at 2.01 Å, 1 imidazole nitrogen ligand at 1.95 Å, and a Zn-Zn interaction at 3.98 Å [110]. The structural changes in the metal sites when another metal binds at the alternate site in HypB are subtle but the XAS studies suggest an allosteric recognition mechanism may be present in HypB similar to that seen in HypA.

**Figure 10.**
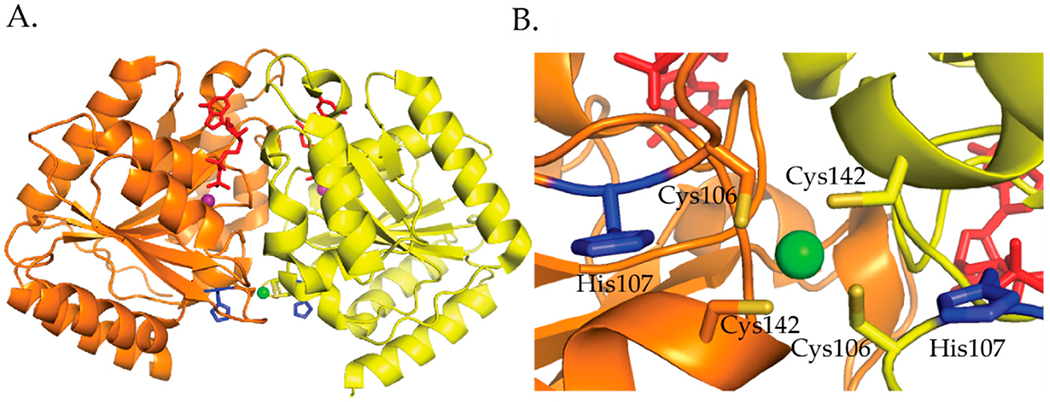
(**A**) Crystal structure of *H. pylori* HypB (PDB ID 4LPS) [114]. Prior to crystallization, the protein was incubated with Guanosine 5′-*O*-(3-thiotriphosphate) (GTPγS) and magnesium chloride before adding nickel chloride [114]. The protein monomers are colored orange and yellow, guanosine diphosphate (GDP) molecules are shown as red spheres, the magnesium ions are shown as purple spheres, and the nickel ion is shown as a green sphere, (**B**) Close-up of the bridging G-domain metal site with a nickel ion bound. (This figure was adapted from Sydor et al [114]. The research was originally published in the Journal of Biological Chemistry. Sydor, A.M.; Lebrette, H.; Ariyakumaran, R.; Cavazza, C.; Zamble, D.B. Relationship between Ni(II) and Zn(II) coordination and nucleotide binding by the *Helicobacter pylori* [NiFe]-hydrogenase and urease maturation factor HypB. *J. Biol. Chem.* **2014**, *289*, 3828–3841. © the American Society for Biochemistry and Molecular Biology *or* © the Author(s)).

*H. pylori* HypB binds Ni(II) ions at the G-domain with a 1:1 stoichiometry and a micromolar affinity determined by Ni(II) ion titrations monitored by UV-vis [106]. Competition metal binding studies involving the metal chelator Mag-fura 2 determined a K_d_ for Ni(II) of 150 nM and 1.2 nM for Zn(II) [111]. Similar to *E. coli* HypB, *H. pylori* HypB binds zinc tighter than nickel in the G-domain [109,111]. Mutagenesis studies involving the conserved residues Cys106 and His107 determined that these residues are important for Ni(II) and Zn(II) binding [111]. The C106A and H107A mutations disrupted Ni(II) binding and weakened Zn(II) binding by 2 orders of magnitude [111]. These results suggest that the coordination spheres of Ni(II) and Zn(II) are not identical but some of the ligands used to bind nickel also bind zinc [111]. *E. coli* HypB GTP hydrolysis is slow [114]. Ni(II) binding to HypB promotes dimerization and has no effect on GTPase activity while zinc binding does not promote dimerization and inhibits GTPase activity [111]. The crystal structure of *H. pylori* HypB with Ni(II) and GDP bound shows that the nickel is ligated by four cysteines in a square planar geometry and that this metal binding site bridges the HypB dimer (Figure 10) [114]. Studies done by Sydor et al. demonstrate that the nucleotide bound state of the protein influences the coordination environment of the nickel ion [114]. These studies suggest that H107 is a nickel ligand in the absence of nucleotide binding to the protein [114]. Additionally, Cys142 was identified as an important residue for coupling metal binding to *H. pylori* HypB with GTPase activity [114].

HypB proteins have also been crystallized from *Methanocaldococcus jannaschii* (*M. jannaschii*) with Zn(II)) and (GTPγS) bound [113]. The protein was purified and incubated in buffer containing zinc chloride and crystallized from a solution containing GTPγS [113]. *T. kodakarensis* with ADP bound [112], and *Archeoglobus fulgidus* (*A. fulgidus*) in the nucleotide free apo form [119]. *T. kodakarensis* hypB is the only HypB protein discussed in this review is an ATPase. *T. kodakarensis* HypB is a member of the Mrp/MinD family of ATPase-type HypB [112]. All four HypB crystal structures show that in HypB there is a homodimer nucleotide-binding site near the dimer interface. Surprisingly, a nickel binding site was not identified for *T. kodakarensis* HypB, which suggest that it functions different from the other HypBs that have been characterized [112]. The HypB crystal structure from *M. jannaschii* revealed that there are two metal binding sites located in the G domain [113]. Like *H. pylori* HypB, the metal binding site bridges the dimer interface [113,114]. Additionally, the location of the nucleotide binding site in *M. jannaschii* is almost identical to that of *H. pylori* HypB [114]. In the crystal structure two zinc ions are bound in two unique sites that are bridged by a cysteine residue (Cys95). [113] One zinc ion is coordinated by three cysteines from one monomer (Cys95 and Cys127), a cysteine from another monomer (Cys95) and a water molecule. [113]. The second zinc ion is coordinated by two cysteine residues (Cys95 and Cys127), histidine (His96) and a water molecule [113]. The structure also revealed that GTPγS binds at the dimer interface [113].

Studies involving *A. fulgidus* and *E. coli* HypB determined that the dimerization of HypB proteins is important for [NiFe]-hydrogenase activity [119,120]. Studies by Cai et al. on *E. coli* HypB determined that Leu242 and Leu246 are important for dimerization of the protein [120]. The residues correspond to Leu171 and Val175 in *M. janaschii* HypB. The crystal structure of *M. Jannaschii* showed that Leu171 and Val175 are located in a hydrophobic patch at the dimer interface [113]. Mutant Leu242A and Leu246A *E. coli* HypB proteins could still bind metal and hydrolyze GTP, however, they could not dimerize resulting in a decrease [NiFe]-hydrogenase activity (half of the {NiFe}-hydrogenase activity that was observed with the wild-type HypB protein) [120]. This result is also supported by work done on *A. fulgidus. A. fulgidus* HypB is a monomer in the apo and the GDP-bound forms but the GTP bound form of the protein is a dimer [119]. This dimer was not observed in a Lys148A (Lys224 in *E. coli*) mutant *A. fulgidus* hypB though the protein could still bind GTP. It was shown that a K22A mutant HypB protein could not recover [NiFe]-hydrogenase activity in a Δ*hypB E. coli* strain [119].

HypB proteins with an N-terminal histidine rich region that are capable of binding several nickel ions have been characterized from *Bradyrhizobium japonicum* (*B. japonium*) and *R. leguminosarum* [121–123]. It has been suggested that these histidine rich tails play a role in nickel storage and buffering [122,124], *B. japonicum* HypB has 24 histidine residues located near the N-terminus. The protein binds 9 Ni(II) per monomer with a K_d_ of 2.3 μM [121]. Other metal ions, including Zn(II), Cu(II), Co(II), Cd(II), and Mn(II) can bind to the protein [121]. *R. leguminosarum* HypB has seventeen histidine residues near the N-terminus and binds four Ni(II) ions per monomer [123]. Other metal ions like Co(II), Cu(II), and Zn(II) can compete with Ni(II) for binding to the protein [123]. The significance of these histidine rich regions in some HypB proteins is unknown but it has been suggested that they may be involved in nickel storage [17].

### 3.3. SlyD

**Figure 11.**
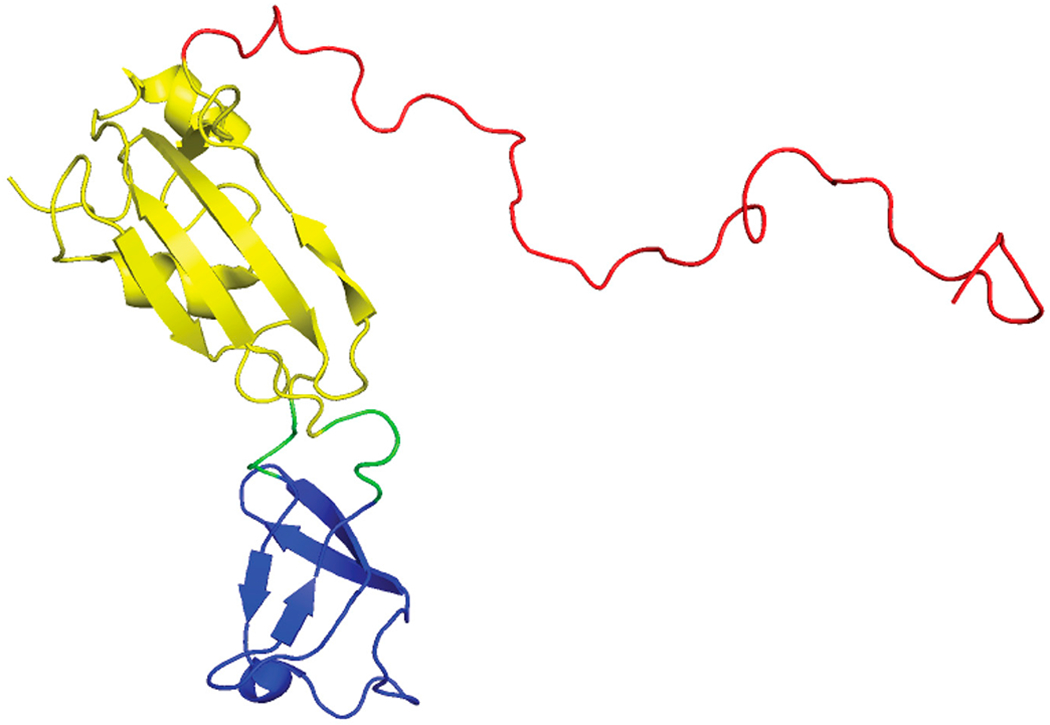
NMR structure of *E. coli* SlyD (PDB ID 2KFW) [126]. The IF domain is colored blue, the PPIase domain is colored yellow, and the unstructured C-terminal metal-binding tail is colored red.

SlyD is a member of the FK506-binding protein (FKBP) family of peptidyl-prolyl cis/trans isomerases [125]. The NMR structures of SlyD from *E.coli* show that the protein is a two-domain protein: a peptidyl-prolyl isomerase (PPIase) domain and the “inserted in the flap” (IF) domain (Figure 11) [126,127]. Prolyl isomerases are involved in numerous biological functions including catalyzing the cis-trans isomerization of proline peptide bonds, which is a crucial step in the folding pathway of some proteins [125,128]. However, in *E. coli* the isomerase function of SlyD is nonessential in [NiFe]-hydrogenase maturation [129]. In *E.coli* it was determined that SlyD is important for [NiFe]-hydrogenase activity as a *slyD* mutation resulted in a 50% reduction in H_2_ production in cultures during exponential phase growth [16]. *E. coli* SlyD has an unstructured C-terminal metal-binding tail (Figure 11), which is rich in metal binding residues: histidines, cysteines, aspartates, and glutamates [17]. The C-terminal tail is variable among SlyDs [126,127,130–132], *E. coli* SlyD can bind up to 7 Ni(II) per protein with submicromolar (K_d_ < 1.8 μM) affinity [133], which reversibly inhibits PPIase activity [134]. A precise model of the Ni(II) site in SlyD could not be determined by XAS studies as there are multiple nickel sites in the protein that had different geometries and coordination numbers [133]. Based on the ability of SlyD to bind multiple metal ions, it is thought that SlyD contributes to Ni(II) storage and is a possible source for Ni(II) ions during [NiFe]-hydrogenase assembly in *E. coli* [15,135].

*H. pylori* SlyD also has a histidine and cysteine rich C-terminal tail that binds 2.4 Ni(II) per protein or 3.3 Zn(II) ions per protein with K_dS_ of 2.74 and 3.79 μM, respectively [132]. A SlyD homolog from *Thermus thermophilius* (*T. thermophilius*) that does not contain the extended cysteine and histidine rich C-terminal tail seen for *E. coli* and *H. pylori* SlyD has a metal binding site composed of three histidines [130]. The crystal structure shows that either Ni(II) or Zn(II) is bound by the conserved sequence (HGHXaaH) [130]. A truncated *E. coli* SlyD protein, SlyD155, was created to resemble the *T. thermophilius* SlyD protein. SlyD155 was able to bind a single nickel ion with a K_d_ of 65 nM and an octahedral geometry with 2–3 imidazole ligands from His149, His151, and His153, and 3–4 N/O-donors, but could not activate [NiFe]-hydrogenase activity in vivo [129].

### 3.4. UreE

UreE delivers nickel to the UreABC-UreDFG complex (Figure 2) [28]. UreE from *K. aerogenes* is a homodimer that binds six nickels per homodimer with a K_d_ of ~9.6 μM [136]. XAS studies determined that UreE binds nickel in different sites. The average Ni(II) site determined for a Ni(II)UreE sample prepared by mixing UreE with approximately three equivalents of Ni(II) per dimer is pseudo-octahedral with six N/O donors of which three to five are histidine ligands [136]. Most of the nickel is bound at the histidine rich C-terminal tail (HGHHHAHHDHHAHSH) [28,137]. *H. pylori* and *Sporosarcina pasteurii* (*S. pasteurii* formerly *Bacillus pasteurii* [138]) lack this histidine rich tail. The *H. pylori* UreE is a homodimer that binds one Ni(II) or Zn(II) per dimer with a K_d_ of 0.15 and 0.49 μM, respectively [139,140]. The nickel site is six-coordinate and features four histidine ligands while the zinc site is five-coordinate with two or three histidine ligands [141]. *S. pasteurii* UreE binds two Ni(II) per dimer with an overall K_d_ of 35 μM [142]. The nickel site is also six-coordinate and features each Ni(II) ion bound by an average of two histidine and 4 N/O residues [142,143]. Studies done by Brayman, Hausinger, and Colpas show that the histidine rich C-terminal tail of *K aerogenes* UreE is not necessary for UreE to deliver nickel to urease [144,145]. A H144* mutant UreE from *K. aerogenes* was generated by removing the last fifteen residues, which includes ten histidine residues [144]. Like the wild-type protein, the H144* UreE is a homodimer, but it bound two Ni(II) per dimer versus the six Ni(II) per dimer, which was observed for the wild-type protein [146]. Urease activity in cells containing H144* UreE was similar to that of cells with the wild-type protein [144].

Cd(II), Co(II), Zn(II) and Cu(II) ions are able to compete with Ni(II) ions for the nickel binding site in H144* UreE [144]. These metals bind to the protein with stoichiometry of two metal ions per dimer using different ligands and with different geometries [146]. The two Ni(II) ions bind with peudo-octahedral geometry and the two Ni(II) sites are spectroscopically distinguishable as one Ni(II) site binds one less histidine residue than the other [146,147]. Equilibrium dialysis experiments determined that Ni(II) bound with K_d_s of 47 μM and 1.45 μM [147]. ITC experiments determined that the two Ni(II) ions bind with a K_d_ of 1.6 nM but do not distinguish between the two Ni(II) binding to UreE [148]. Similar to *E. coli* NikR, a difference in the K_d_s determined using two different techniques is observed. The two Cu(II) ions bind to H144* UreE with tetragonal geometry with each Cu(II) being coordinated by two histidine ligands and one Cu(II) ion is bound by a cysteine residue, Cys79 [146,147]. Like Ni(II), Co(II) ions adopt pseudo-octahedral geometry, and the two cobalt sites differ in the number of histidine ligands that coordinate to each Co(II) ion [146]. The metal binding ligands were determined using a combination of mutagenesis and urease activity studies coupled with metal binding studies involving equilibrium dialysis, and UV-visible, EPR, and NMR spectroscopies [147]. It was suggested that one Ni(II) or Co(II) is bound by His96, His112, and 1 N/O-donors from each monomer forming a six-coordinate metal site. The second Ni(II) or Co(II) is bound by His110, and two N/O-donors from each monomer forming a six-coordinate metal site [147]. Proton NMR studies suggest that the nickel and cobalt sites differ in the number of histidine ligands at each site. The first nickel site has one histidine and the second has two histidines; the number of histidine residues are reversed for the cobalt site [146]. It is important to note that the metal binding ligand His96 is conserved among UreE proteins [147] and was shown to be important for urease activation both in vitro [149] and in vivo [147]. Proton NMR studies suggest that the nickel and cobalt sites differ in the number of histidine ligands at each site. The first nickel site has one histidine and the second has two histidines, the number of histidine residues are reversed for the cobalt site [146].

The *K. aerogenes* H144* crystal structure showed that the protein binds three Cu(II) ions per dimer [150]. This crystal structure was solved from apo H144* crystals that were soaked with copper(II) sulphate [150]. One copper is bound in between the two monomers by two His96 residues, one from each monomer. The other two coppers are each coordinated by His110 and His112 within each monomer. The differences in the metal binding stoichiometries of Cu(II) binding to H144* UreE determined by Colpas et al. [146,147] in the crystal structure are explained by ITC experiments. These studies determined that the number of Ni(II) and Cu(II) ions bound per dimer is dependent on the concentration of the protein. At low concentrations (<10 μM), the dimeric H144* UreE binds 2 Ni(II), or Cu(II) ions per dimer but at higher concentrations, 25 μM, binds 3 Ni(II) or Cu(II) per dimer [148]. The ITC data also showed evidence of the formation of a tetramer (dimer of dimers) at higher concentrations [148]. Additionally, Cu(II) binds tighter to *K. aerogenes* than Ni(II) but Ni(II) binding is enthalpically favored.

The first crystal structure of UreE from *S. pasteurii* shows that Zn(II) binds at the interface of two dimers and is bound by four histidine residues (His 100 equivalent to His96 from *K. aerogenes*) from each monomer [151]. The protein crystals were obtained from UreE protein that copurified with Zn(II) ions [151]. A second set of crystal structures of *S. pasteurii* UreE bound to Ni(II) or Zn(II) show that the Ni(II) is coordinated by His100 in a site that is consistent with octahedral geometry while the Zn(II) site adopts pseudo-tetrahedral geometry and is bound by His9 and Asp12 from two different dimers [138]. In this study, the protein crystals were obtained from apo UreE that was incubated with nickel sulphate or zinc sulphate [138]. Crystallographic studies coupled with ITC suggest that there is a high affinity and a low affinity nickel site located at the C-terminus of the protein. The high affinity site features His100 as Ni(II) ligands and the low affinity site features His145 or His147 [138,142]. It has been suggested that the low affinity site funnels nickel to the high affinity site, which can bind either Ni(II) or Zn(II), and could be involved in UreE-UreG interactions [138].

*H. pylori* UreE has been crystallized in the apo, Ni(II) bound, Cu(II) bound, and Zn(II) bound forms [141,152]. The structures determined by Shi et al. show the apo protein as a dimer and the metal bound Cu(II) and Ni(II) forms of the protein as a tetramer, a dimer of dimers [152]. It is unclear what the exact source of the Cu(II) and Ni(II) ions is in these structures [152]. The Cu(II) and Ni(II) UreE crystals resulted from an attempt crystallize the UreE–HypA and the UreE-UreG complex, respectively [152]. However, in the structures obtained by Banaszak et al. the protein exists as a dimer in the apo, Zn(II) and Ni(II) forms [141]. These ZnUreE and NiUreE structures were obtained from apo UreE that was incubated with either zinc sulfate or nickel sulphate and crystallized [141]. Both sets of structure show that His102 (equivalent to His96 and His100 in *K. aerogenes* and *S. pasteurii, respectively*) is involved in binding the metal ion [141,152]. *H. pylori* UreE has a single His residue, His152, on the C-terminus. Mutagenesis studies coupled with ITC experiments determined that His152 is a ligand for Zn(II) and not Ni(II) [140]. It was also determined by ITC experiments that His102 is a ligand for both Ni(II) and Zn(II) [140].The crystal structure and the XAS data revealed that the *H. pylori* Ni(II) site is pseudo-octahedral and the Zn(II) site is tetrahedral and both metals are bound by His102 and His152 [141]. Although there is a difference in the ligands bound to Ni(II) in the H152A UreE compared to wildtype, Banaszak et al. suggests that mutating His152, which may be a weakly coordinated residue, results in a rearrangement in the metal site [141].

## 4. Protein-Protein Interactions

The maturation of [NiFe]-hydrogenase and urease is a highly choreographed process that involves several accessory proteins. Protein–protein interactions between the metallochaperones HypA, HypB and SlyD have been observed experimentally, however, a ternary complex has not been detected [153]. Work done by Khorasani-Motlagh et al. suggests that HypB is the central component of nickel delivery to [NiFe]-hydrogenase as it interacts with both SlyD and HypA, individually [153]. Based on cyclic voltammetry (CV) and electrochemical impedance spectroscopy (EIS) measurements, the relative affinities of the HypB complexes are on the order of HypB–SlyD > HypB–HypA > HypA–SlyD [153].

**Figure 12.**
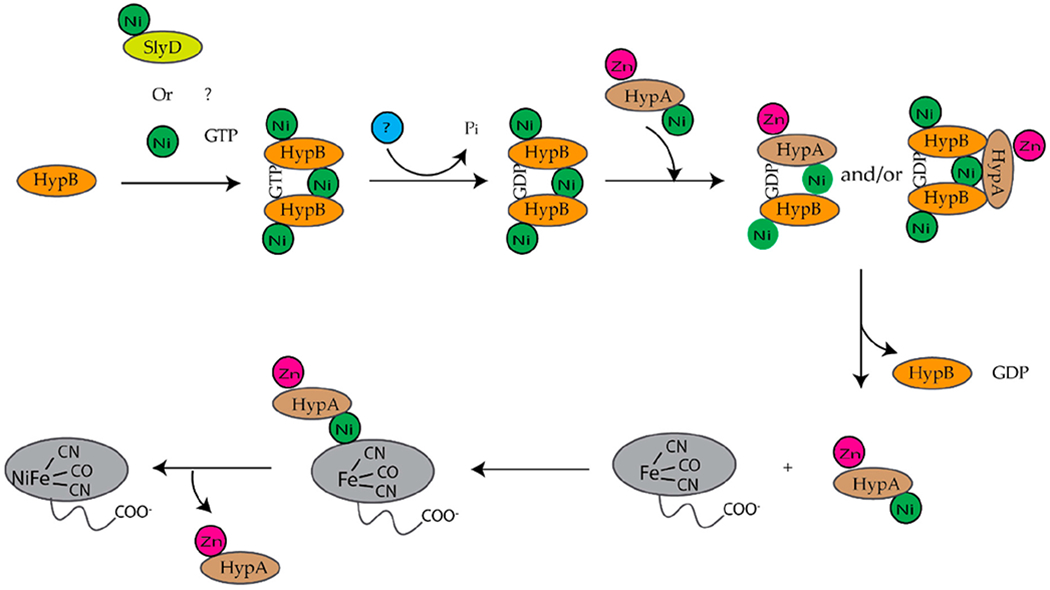
Model of the protein complexes required for the insertion of nickel into the large [NiFe]-hydrogenase precursor protein in *E. coli* [17,117,135]. Monomeric HypB dimerizes when it binds to GTP and then binds to nickel from SlyD or some other source. GTP hydrolysis occurs, which weakens the affinity of HypB for nickel and promotes the formation of a complex between NiGDPHypB and ZnHypA. Nickel is transferred from NiGDPHypB to ZnHypA. NiZnHypA then delivers nickel to the large [NiFe]-hydrogenase precursor protein.

HypB and SlyD form a complex in both *E. coli* [15,129,154] and *H. pylori* [155]. In both bacteria, SlyD enhances the GTPase activity of HypB [135,156]. *E. coli* SlyD also forms a complex with a Strep-tagll variant of the large subunit of [NiFe]-hydrogenase 3, HycE [157]. Additionally, it was determined that the C-terminal tail of SlyD is important for stimulating nickel release from HypB [129]. HypB also forms a heterodimer with HypA in *H. pylori* [95,106], and *E.coli* (Figure 12) [94]. Nickel is transferred from the G-domain of HypB to HypA and the rate of transfer increases significantly in the presence of GDP [102]. The transfer of nickel is also more efficient when HypA and HypB from a complex [102]. Once HypA is loaded with nickel the HypAB protein complex dissociates (Figure 12) [117]. In *E. coli,* HypA can form a complex with HycE in the absence of HypB or SlyD [158]. It is thought that HypA and HypB preassemble before reaching HycE as both proteins can interact in the absence of HycE [102,106,158]. Deletion of the *hypA* gene prevents HypB from interacting with HycE, which suggests that HypA serves as a scaffold for HypB to dock to the large [NiFe]-hydrogenase precursor protein [107,158].

**Figure 13.**
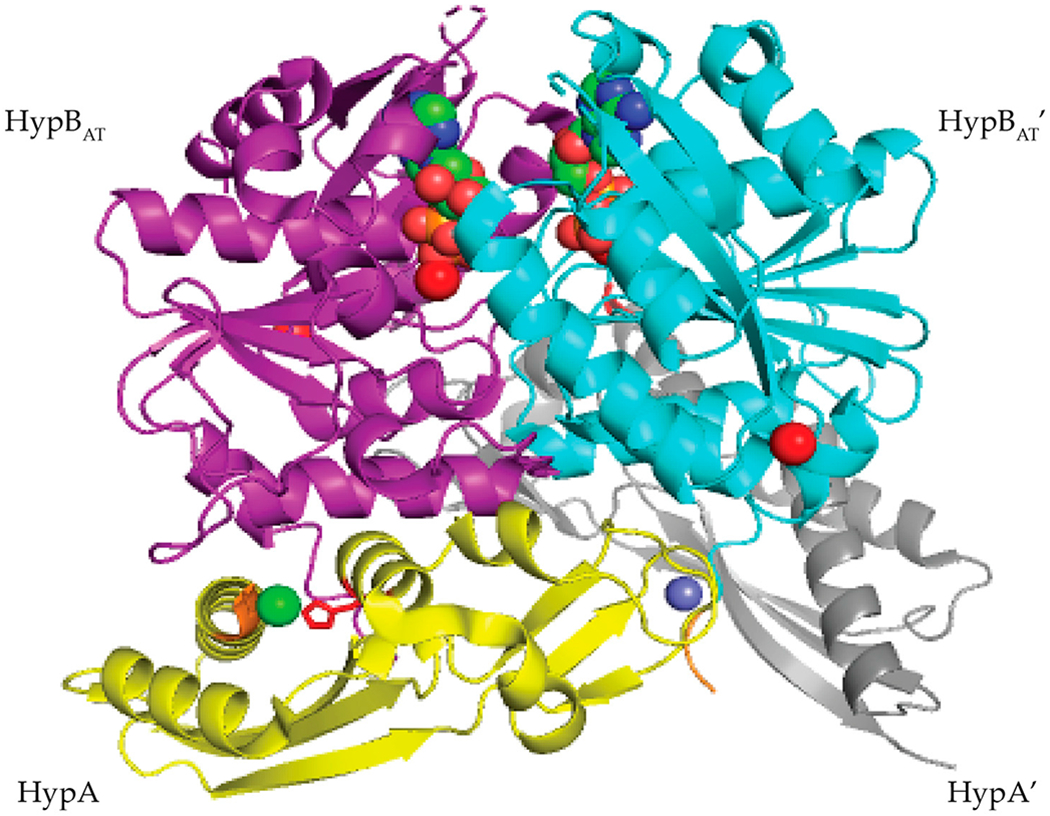
Crystal structure of the transient HypAB complex from *T. kodakarensis* (PDB ID 5AUN) [101]. The protein was crystallized from a solution containing equal molar concentrations of *T. kodakarensis* HypA and HypB in the presence of ATPγS and nickel chloride. The HypA monomers (shown in yellow and gray) bind on either site of the HypB dimer (shown in purple and cyan). The nickel ion is shown as a green sphere and is coordinated by the MHE motif (colored orange) and His98 (colored red) of HypA. The zinc ion is shown as a slate sphere, the magnesium ion as a red sphere, and the ADP molecules are shown as red, green, and blue spheres. (This figure is adapted from Watanabe, S.; Kawashima, T.; Nishitani, Y.; Kanai, T.; Wada, T.; Inaba, K.; Atomi, H.; Imanaka, T.; Miki, K. Structural basis of a Ni acquisition cycle for [NiFe] hydrogenase by Ni-metallochaperone HypA and its enhancer. *Proc. Natl. Acad. Sci. USA* **2015**, *112*, 7701–7706. Copyright (2015) National Academy of Sciences).

In *T. kodakarensis*, HypA enhances the ATPase activity of HypB threefold [101]. Similar to *E. coli* HypAB interactions, HypAB interactions in *T. kodakarensis* are regulated by nucleotide hydrolysis. HypA and HypB form a heterotetrametric structure containing two HypA and two HypB in the presence of ATP (Figure 13) [101]. The HypAB complex binds Ni(II) with nanomolar affinity [101]. The crystal structure shows that the two HypA molecules are bound to the opposite surface of the ATP-binding site of the HypB dimer (Figure 13) [101]. Complex formation between HypA and HypB results in conformational changes that create a new nickel binding site with nanomolar affinity as determined by ITC (Figure 13) [101]. In the HypAB complex, His98 from HypA moves closer to the N-terminus of the protein forming a new nickel site involving His98 and the N-terminal MHE motif (Figure 13) [101]. The Ni(II) ion is bound in a four-coordinate, distorted square planar geometry and is coordinated by the N-terminal amine, the amide nitrogen and side chain of His2, and the side chain of His98 [101].

**Figure 14.**
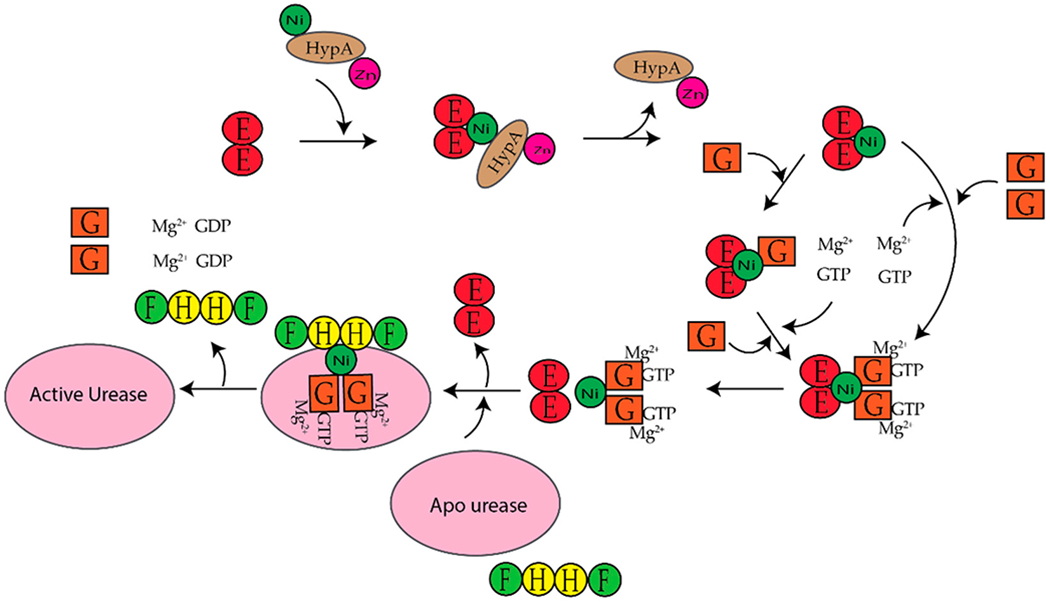
Model of the protein complexes required for the insertion of nickel into *H. pylori* urease [103,160,161]. NiZnHypA forms a complex with UreE_2_ and transfers nickel to UreE_2_–NiUreE_2_ will form a NiUreE_2_–UreG_2_ complex either with UreG_2_ or with 2 UreG in the presence of magnesium ions and GTP. A NiUreE_2_–UreG_2_ complex with magnesium ions, GTP and UreG_2_ is formed and nickel is transformed from UreE_2_ to UreG_2_–UreG_2_ forms a complex with UreF–UreH and apo urease. GTP is hydrolyzed by UreG and nickel is inserted into apo urease in the presence of potassium bicarbonate (not shown) and ammonium bicarbonate (not shown). UreF, UreH, and UreG dissociate from the Ni-urease enzyme.

HypA has also been shown to form a complex with UreE that results in the formation of a new nickel site [96,103,159]. In *H. pylori,* HypA-UreE interactions are essential for urease maturation [31]. Crosslinking, static light scattering, and ITC studies show that the UreE dimer binds HypA using residues located in the C-terminus of the protein to form a hetero-complex, HypA-UreE (Figure 14) [103,159]. The dissociation constant for apo ZnHypA or NiZnHypA to apo-UreE_2_ is 1μM at pH 7.2 [159]. Similarly, the dissociation constant of ZnHypA to ZnUreE_2_ is also 1 μM [159]. However, the interactions between HypA and UreE_2_ were weakened between Zn-HypA or NiZnHypA and Ni-UreE_2_ at pH 7.2 [159].

At both pH 7.2 and pH 6.3 *H. pylori* UreE_2_ binds Ni(II) one order of magnitude tighter than *H. pylori* HypA [96,97,103,140]. It has been determined that Ni(II) is transferred from HypA to UreE**2** (Figure 14) [159]. ITC studies and fluorometric studies revealed that Ni(II) binding to ZnHypA·ApoUreE_2_ results in at least two distinct isotherms with micromolar and nanomolar affinity at both pH values [103]. The micromolar binding is similar to that seen for Ni(II) binding to apoUreE_2_ [103]. Nanomolar binding was also observed when NiZnHypA was titrated into apoUreE_2_ suggesting that a new high affinity nickel binding site is formed when Ni(II) is added to ZnHypA·UreE_2_ complex [103]. This result was further supported by metal binding experiments conducted using L2*HypA mutant protein. It was determined that ZnL2*HypA binds apoUreE_2_ similar to wild-type HypA with micromolar affinity and that ZnL2*HypA has a weaker affinity for Ni(II) than the wild-type HypA protein [96,103]. Additionally, titrating ZnL2*HypA·apoUreE_2_ with Ni(II) at pH 7.2 resulted in a single isotherm [103].

In *K. aerogenes* the urease accessory proteins UreD, UreF, UreG, and UreE bind sequentially to the urease enzyme (Figure 1). First, UreD forms a complex with apo-urease, UreABC–UreD [28]. In the presence of UreD, UreF forms a UreABC–UreDF complex [28,162] and UreG forms a complex with UreABC–UreDF [28,163]. Additionally, the UreDFG complex can bind directly to UreABC [28]. Finally, UreE delivers Ni(II) to the UreABC–UreDFG [149]. In *H. pylori* two different UreE–UreG complexes have been observed (Figure 14). The first complex is composed of two monomers of *H. pylori* UreG bound to one *H. pylori* UreE dimer (UreE_2_–UreG_2_) with a K_d_ of 4.0 μM [140,160]. A second UreE–UreG complex has been observed that features a UreE dimer with a UreG monomer (UreE_2_–UreG) (Figure 14) [160]. It was determined that UreE accepts Ni(II) from HypA and the UreE_2_–UreG_2_ complex facilitates Ni(II) transfer from UreE to UreG and enhances GTP binding [160].

Several studies have been done on individual proteins and protein–protein interactions between the Ni(II) chaperones and accessory proteins. These studies have led to models for nickel insertion into *E. coli* [NiFe]-hydrogenase (Figure 12) and *H. pylori urease* (Figure 14). For nickel insertion into *E. coli* [NiFe}-hydrogenase involves the dimerization of HypB when it binds to GTP and nickel. GTP is hydrolyzed to GDP, and NiGDPHypB forms a complex with ZnHypA. The stoichiometry of the HypA-HypB complex is unknown [117]. Nickel is then transferred from the G-domain nickel site of HypB to N-terminal nickel site of ZnHypA, and NiZnHypA forms a complex with and transfers nickel to the large [NiFe]-hydrogenase precursor protein. For nickel insertion into *H. pylori* urease activation involves NiZnHypA delivering nickel to UreE_2_. UreE_2_ binds to UreG_2_ and transfers nickel to UreG_2_. NiUreG_2_ form a complex with UreH_2_F_2_ and apo urease. GTP hydrolysis occurs and nickel is delivered to urease followed by the dissociation of UreF, UreE, and UreG from the active urease enzyme.

## 5. Conclusions

Over the past 20 years significant progress has been made towards elucidating the role that nickel plays in the proper functioning of metalloregulators and chaperones. Multiple themes have emerged from the studies conducted on nickel metalloregulators and chaperones regarding nickel binding and protein allostery. The proteins discussed in this review bind nickel in one of three different geometries: four-coordinate square planar, four-coordinate tetrahedral, or six-coordinate octahedral geometry. Four of the proteins, RcnR, NmtR, HypA, and *E. coli* HypB, utilize the N-terminal amine to coordinate the nickel ion. The Ni(II)-responsive metalloregulators: RcnR, DmeR, and KmtR are also Co(II)-responsive.

Many of the metalloregulators discussed in this review can bind other first row transition metals but only cognate metal, nickel and in some cases cobalt, binding results in the allosteric regulation of DNA binding. NikR proteins have been studied extensively using a combination of NMR, X-ray crystallography, XAS, and metal binding studies. These studies determined that NikR can bind to the first-row transition metals, Co(II), Ni(II), Cu(II), and Zn(II) with various geometries [47,57,59]. The binding affinities clearly follow the Irving Williams series, yet NikR is a Ni(II) responsive metalloregulator [59]. These results suggest that only when nickel binds to NikR forming a four coordinate square planar complex using 3 N_Imidazole_ ligands and 1 S-dnonors does the correct structural changes occur in the protein that favors DNA binding [57]. These results are corroborated by studies done in RcnR, NmtR, and InrS where nickel binding disfavors DNA binding. Collectively, these results support the theme that metal responsiveness is most closely linked to coordination number and ligand selection [36,164]. Protein allostery is also observed when Ni(II) binds to *H. pylori* HypA [97,104] and *E. coli* HypB [110].

Another theme that emerges from the work discussed in this review is that sequence similarities between the metalloregulators are not sufficient to determine the metal selectivity of metalloregulators. For example InrS possesses all the ligands that bind Cu(I) in *M. tuberculosis* CsoR, however, InrS uses these ligands plus an additional His ligand to bind nickel [63,77]. Interestingly, both RcnR and InrS belong to the same family of metalloregulators and thus have a similar protein fold but they coordinate nickel with a different ligand set and geometry. *E. coli* RcnR coordinates nickel in a six-coordinate octahedral site [70,72,73]. InrS utilizes a different ligand set to coordinate nickel in four-coordinate square planar geometry [79]. Why has nature designed two metalloregulators that carryout similar functions with the same fold but different metal binding geometries and ligand sets? It will be interesting to see how DmeR binds nickel. A similar situation exists for the two Ni(II) and Co(II) regulators from *M. tuberculosis,* NmtR and KmtR [84–86,89].

HypA, HypB, and SlyD proteins have been characterized from various organisms including *E. coli* and *H. pylori* and the metal site structures have been elucidated. Variations in the number of metal binding sites and metal ligands among homologous proteins from different organisms have been observed. The reasons for these variations are unknown but it may be linked to differences in intracellular nickel concentrations or the nickel requirements of the organism [17] as well as the various protein-protein interactions that occur in the shuttling of nickel from one protein to another. All HypA proteins have two metal binding sites, an N-terminal nickel site and a zinc site in a loop region with 2 CXXC motifs. However, both the flanking His residues, His79 and His95, in *H. pylori* HypA are not conserved in all HypA proteins, some have one or none [97]. Mutating any of the Cys or His residues resulted in a HypA protein that no longer sensed nickel binding or pH changes [97]. The variations in the number of flanking His residues in HypA proteins suggest that if nickel binding and pH changes are communicated in other HypA proteins, they do not result in similar structural changes observed for *H. pylori* HypA. A regulatory role for the *H. pylori* zinc site has been proposed [33,97,104,105]. It has been suggested that changes in the zinc site may affect interactions between HypA and other nickel proteins [33,97] and may affect the delivery of nickel to [NiFe]-hydrogenase and urease [33,105]. Studies involving mutant *hypA* strains have demonstrated that all the cysteine residues in the zinc site are important for [NiFe]-hydrogenase and urease activity in *H. pylori* [33,105]. The His residues may play a role in protein–protein interaction as they are not required for [NiFe]-hydrogenase and urease activity [33,105].

The number of metal binding sites varies in HypB proteins. All GTPase HypB proteins have a nickel site located in the G domain that binds either nickel or zinc. This site has a lower affinity for nickel than zinc but is involved in transferring nickel to HypA [109,111,117]. A N-terminal high affinity site has been characterized for *E. coli* HypB [109]. The exact role of this site still needs to be elucidated [109]. Leach et al. have suggested that that the N-terminal nickel site plays a structural and/or regulatory role [109]. They have also suggested that it could be a source of nickel for [NiFe]-hydrogenase when nickel concentrations in the cell are limited [109]. Some HypB proteins have histidine rich regions that bind multiple nickel ions that may be involved in nickel storage. Studies done by Sydor et al. on *H. pylori* HypB showed that there is a link between nucleotide binding and the ligands used to coordinate nickel. When GDP is bound to the protein, *H. pylori* HypB binds nickel with 4 S-donors from four cysteine residues, however, in the absence of nucleotide bound to the protein His107 ligates the nickel [114]. The importance of this change is not fully understood but it may play a role in hydrogenase and urease maturation [114].

SlyD is important for [NiFe] hydrogenase maturation [16] and is known to form a complex with HypB, [15,129,154,155]. However, the details of the interaction between SlyD and HypB still need to be determined. The importance of histidine rich C-terminal tails vary in [NiFe]-hydrogenase and urease maturation. Both *H. pylori* SlyD [132] and *K. aerogenes* UreE [136] have histidine rich C-terminal tails. It was shown that the histidine rich tail in SlyD is essential for [NiFe]-hydrogenase maturation [129], but the C-terminal tail of UreE is not necessary for urease maturation [144]. All UreE proteins characterized use a single conserved His residue and other residues to bind nickel in octahedral or pseudo octahedral geometry at the dimer interface. *S. pasteurii* UreE, like *E. coli* NikR and *E. coli* HypB has a high and low affinity nickel binding site [138,142]. It is thought that the low affinity site funnels nickel to the high affinity site, and the high affinity site is involved in UreE–UreG interactions [138].

The bioinorganic chemistry of nickel metalloregulators and chaperones is fascinating. Numerous studies have been conducted on individual chaperones and accessory proteins involved in [NiFe]-hydrogenase and urease maturation but some details regarding the sequential protein-protein interactions that are necessary to deliver nickel to [NiFe]-hydrogenase and urease still need to be elucidated. How does HypB get nickel? What is the exact role of SlyD in the delivery of nickel to hydrogenase? What role does HypB play in urease maturation? In *E. coli* what is the oligomeric state of the HypA–HypB complex? What favors HypA delivering nickel to [NiFe]-hydrogenase versus UreE and vice versa? What ligands compose the HypA-UreE metal binding site? How does UreG deliver nickel to urease? What is the role of UreD? How does UreF stimulate the GTPase activity of UreG?

For *H. pylori*, studies involving animal models have determined that the pathogenicity of *H. pylori* is dependent on both [NiFe]-hydrogenase and urease [26,165,166]. Additional studies done by Blum et al. determined that urease and not [NiFe] hydrogenase is responsible for acid resistance in *H. pylori* [105]. To date there are no mammalian enzymes that utilize nickel [1], therefore, drug therapies targeting proteins involved in nickel ion homeostasis could be used to treat *H. pylori* infections [24]. Prior to developing such therapies, an understanding of how nickel is bound by the various proteins in the cells and the details of protein-protein interactions involved in delivering nickel to [NiFe]-hydrogenase and urease in *H. pylori* is crucial.

## Supplementary Material

Supplementary Material
