# Supplementary material for: Nickel Metalloregulators and Chaperones
Source: Inorganics (Basel). Author manuscript; Available in PMC 2020 Mar 4. (PMC7055735; doi:10.3390/inorganics7080104)
Supplement: Supplementary Material [file NIHMS1562184-supplement-Supplementary_Material.pdf]

Supplementary Materials: Nickel Metalloregulators and Chaperones

Khadine Higgins

Table S1. The metal binding properties of the Ni(II)-responsive metalloregulators.

Protein	Organism	Number of Metal Ions per Functional Unit	K_d	pH [d]	CN #	Metal Site Structure	References
NikR	*E. coli*	4 Ni/tetramer	HA site: 6.8 pM [a]	7.5	4	$NiS(N_{Im})_3$	[1–3]
			HA site: 0.93 pM [a]	7.6			[4]
			HA site: 25 nM, 400 nM [b]	8.0			[5]
		2 Ni/tetramer	LA site: 29 nM [c]	7.5	6	$Ni(O)_6$ or $Ni(N/O)_4(N_{Im})_2$	[2,3,6]
		4 Ni/tetramer	LA site: 700 nM [b]	8.0	ND	ND	[5]
	H. pylori	4 Ni/tetramer	HA site: 12 nM, 125 nM [b]	7.0	4	$NiS(N_{Im})_3$	[7,8]
		10 Ni/tetramer	LA site: 0.5 μM [b]	7.0	ND	ND	[8]
Nur	*S. coelicolor*	4 Ni/tetramer	10 nM, 280 nM [b]	8.0	-	-	[5,9]
RcnR	*E. coli*	4 Ni/tetramer	<25 nM [a]	7.0	6	$NiSNH_2ON_{Im}(N/O)_2$	[10–13]
			17nM, 300 nM [b]	7.0			[5]
		4 Co/tetramer	5 nM [a]	7.0	6	$CoSNH_2(N_{Im})_2(O)_2$	[10–13]
InrS	*Synechocystis*	4 Ni/tetramer	0.02 pM [a]	7.8	4	$Ni(N_{Im})_2(S)_2$	[14,15]
			70 nM, 4.5 μM [b]	7.8			[5]
NmtR	*M. tuberculosis*	2 Ni/dimer	87 pM, 0.14 nM [a]	7.0	6	$NiNH_2(N_{Im})_4O$	[16,17]

The imidazole ligand from histidine is abbreviated as N_{Im}. [a] K_d determined using competition studies. [b] K_d determined by ITC. [c] K_d determined by direct metal titrations. [d] The pH of the buffer used in the metal binding studies. - There is some ambiguity.

Table S2. The metal binding properties of the Ni(II) chaperones.

Protein	Organism	Number of Metal Ions per Functional Unit	K_d	pH [e]	CN #	Metal Site Structure	References
HypA	*E. coli*	1 Ni/dimer	61 μM [c]	7.5	ND	ND	[18]
			75 nM [a]	7.5			[19]
		1 Zn/monomer	0.9 nM [a]	7.5	4	$Zn(S)_4$	[18]
	H. pylori	1 Ni/monomer	~1 μM [b]	7.2	6	$NiNH_2(N)_2O_2N_{Im}$	[20–23]
			58 μM, 1.3 μM [d]	8.25	ND	ND	[24]
		0.5 Ni/monomer	17 μM [b]	6.3	6	-	[21]
		1 Zn/monomer	ND	7.2	4	$Zn(S)_4$	[21]
			ND	6.3	4	$Zn(S)_3N_{Im}$	[21]
HypB	*E. coli*	2 Ni/dimer	HA site: 0.13 pM [a]	7.6	4	$NiNH_2(S)_3$	[25–27]
		2 Ni/dimer	LA site: 12 μM [c]	7.6	6	$NiS(N/O)_4N_{Im}$	[25,26]
		2 Zn/dimer	LA site: 1 μM [c]	7.6	4	$Zn(S)_{2.5}N/OZn(N_{Im})_{0.5}$	[25,26]
	H. pylori	2 Ni/dimer	1.7 μM [c], 0.21 μM [a]	7.4	4	NiS_4 [f]	[28,29]
			150 nM [a],	7.6			[30]
		2 Zn/dimer	1.2 nM [a]	7.6	ND	ND	[30]
	B. japonicum	18 Ni/dimer	2.3 μM [d]	8.3	ND	ND	[31]
SlyD	*E. coli*	7 Ni/monomer	<1.8 μM [a]	7.5	ND	ND	[32]
	H. pylori	2.4 Ni/monomer	2.74 μM [d]	7.6	ND	ND	[33]
		3.3 Zn/monomer	3.79 μM [d]	7.6	ND	ND	[33]
SlyD 155	*E. coli*	1 Ni/Monomer	65 nM [b]	7.5	6	$Ni(N/O)_{3–4}(N_{Im})_{2–3}$	[34]
UreE	*K. aerogenes*	6 Ni/dimer	9.6 μM [d]	7.2	6	$Ni(N/O)_{1–3}(N_{Im})_{3–5}$	[35]
	H. pylori	1 Ni/dimer	0.15 μM [b]	7.0	6	$Ni(N/O)_2(N_{Im})_4$	[36,37]

		1 Zn/dimer	0.49 μM [b]	7.0	5	$Zn(N/O)_2(N_{Im})_2Br$	[36,37]
	S. pasteurii	2 Ni/dimer	35 μM [c]	7.5	6	$Ni(N/O)(N_{Im})_2$	[38,39]
H144* UreE	*K. aerogenes*	2Ni/dimer	47 μM, 1.5 μM [d]	7.5	6	$Ni(N/O)_2(N_{Im})_4$ and $Ni(N/O)_4(N_{Im})_2$	[40]
			1.6 nM [b]				[41]

The imidazole ligand from histidine is abbreviated as N_{Im}. [a] K_d determined using competition studies. [b] K_d determined by ITC. [c] K_d determined by direct metal titrations. [d] Kd determined by equilibrium dialysis. [e] The pH of the buffer used in the metal binding studies. [f] The nickel site when GDP is bound. - There is some ambiguity.

References

1. Chivers, P.T.; Sauer, R.T. NikR repressor: high-affinity nickel binding to the C-terminal domain regulates binding to operator DNA. *Chem. Biol.* **2002**, *9*, 1141–1148.
2. Schreiter, E.R.; Wang, S.C.; Zamble, D.B.; Drennan, C.L. NikR-operator complex structure and the mechanism of repressor activation by metal ions. *Proc. Natl. Acad. Sci. U. S. A.* **2006**, *103*, 13676–13681.
3. Leitch, S.; Bradley, M.J.; Rowe, J.L.; Chivers, P.T.; Maroney, M.J. Nickel-specific response in the transcriptional regulator, *Escherichia coli* NikR. *J. Am. Chem. Soc.* **2007**, *129*, 5085–5095.
4. Wang, S.C.; Dias, A. V; Bloom, S.L.; Zamble, D.B. Selectivity of metal binding and metal-induced stability of *Escherichia coli* NikR. *Biochemistry* **2004**, *43*, 10018–10028.
5. Musiani, F.; Zambelli, B.; Bazzani, M.; Mazzei, L.; Ciurli, S. Nickel-responsive transcriptional regulators. *Metallomics* **2015**, *7*, 1305–1318.
6. Bloom, S.L.; Zamble, D.B. Metal-selective DNA-binding response of *Escherichia coli* NikR. *Biochemistry* **2004**, *43*, 10029–10038.
7. Benini, S.; Cianci, M.; Ciurli, S. Holo-Ni^{2+} *Helicobacter pylori* NikR contains four square-planar nickel-binding sites at physiological pH. *Dalt. Trans.* **2011**, *40*, 7831–7833.
8. Zambelli, B.; Bellucci, M.; Danielli, A.; Scarlato, V.; Ciurli, S. The Ni^{2+} binding properties of *Helicobacter pylori* NikR. *Chem. Commun. (Camb).* **2007**, 3649–51.
9. An, Y.J.; Ahn, B.E.; Han, A.R.; Kim, H.M.; Chung, K.M.; Shin, J.H.; Cho, Y.B.; Roe, J.H.; Cha, S.S. Structural basis for the specialization of Nur, a nickel-specific Fur homolog, in metal sensing and DNA recognition. *Nucleic Acids Res.* **2009**, *37*, 3442–3451.
10. Iwig, J.S.; Leitch, S.; Herbst, R.W.; Maroney, M.J.; Chivers, P.T. Ni(II) and Co(II) sensing by *Escherichia coli* RcnR. *J. Am. Chem. Soc.* **2008**, *130*, 7592–7606.
11. Higgins, K.A.; Chivers, P.T.; Maroney, M.J. Role of the N-terminus in determining metal-specific responses in the *E. coli* Ni- and Co-responsive metalloregulator, RcnR. *J. Am. Chem. Soc.* **2012**, *134*, 7081–7093.
12. Higgins, K.A.; Hu, H.Q.; Chivers, P.T.; Maroney, M.J. Effects of select histidine to cysteine mutations on transcriptional regulation by *Escherichia coli* RcnR. *Biochemistry* **2013**, *52*, 84–97.
13. Carr, C.E.; Musiani, F.; Huang, H.; Chivers, P.T.; Ciurli, S.; Maroney, M.J. Glutamate ligation in the Ni(II)- and Co(II)-responsive *Escherichia coli* transcriptional regulator, RcnR. *Inorg. Chem.* **2017**, *56*, 6459–6476.
14. Foster, A.W.; Patterson, C.J.; Pernil, R.; Hess, C.R.; Robinson, N.J. Cytosolic Ni(II) sensor in cyanobacterium. *J. Biol. Chem.* **2012**, *287*, 12142–12151.
15. Carr, C.E.; Foster, A.W.; Maroney, M.J. An XAS investigation of the nickel site structure in the transcriptional regulator InrS. *J. Inorg. Biochem.* **2017**, *177*, 352–358.
16. Reyes-Caballero, Hermes Lee, Chul Won Giedroc, D.P. *Mycobacterium tuberculosis* NmtR harbors a nickel sensing site with parallels to *Escherichia coli* RcnR. *Biochemistry* **2011**, *50*, 7941–7952.
17. Lee, C.W.; Chakravorty, D.K.; Chang, F.-M.J.; Reyes-Caballero, H.; Ye, Y.; Merz, K.M.; Giedroc, D.P. Solution structure of *Mycobacterium tuberculosis* NmtR in the apo state: insights into Ni(II)-mediated allostery. *Biochemistry* **2012**, *51*, 2619–2629.
18. Atanassova, A.; Zamble, D.B. *Escherichia coli* HypA is a zinc metalloprotein with a weak affinity for nickel. *J. Bacteriol.* **2005**, *187*, 4689–4697.
19. Douglas, C.D.; Ngu, T.T.; Kaluarachchi, H.; Zamble, D.B. Metal Transfer within the *Escherichia coli* HypB–HypA Complex of Hydrogenase Accessory Proteins. *Biochemistry* **2013**, *52*, 6030–6039.

20. Hu, H.Q.; Johnson, R.C.; Merrell, D.S.; Maroney, M.J. Nickel ligation of the N-terminal amine of HypA is required for urease maturation in *Helicobacter pylori*. *Biochemistry* **2017**, *56*, 1105–1116.
21. Herbst, R.W.; Perovic, I.; Martin-Diaconescu, V.; O'Brien, K.; Chivers, P.T.; Pochapsky, S.S.; Pochapsky, T.C.; Maroney, M.J. Communication between the zinc and nickel sites in dimeric HypA: metal recognition and pH sensing. *J. Am. Chem. Soc.* **2010**, *132*, 10338–10351.
22. Spronk, C.A.E.M.; Żerko, S.; Górka, M.; Koźmiński, W.; Bardiaux, B.; Zambelli, B.; Musiani, F.; Piccioli, M.; Basak, P.; Blum, F.C.; Johnson, R. C.; Hu, H.; Merrell, D. S.; Maroney, M.; Ciurli, S. Structure and dynamics of *Helicobacter pylori* nickel-chaperone HypA: an integrated approach using NMR spectroscopy, functional assays and computational tools. *J. Biol. Inorg. Chem.* **2018**, *23*, 1309–1330.
23. Hu, H.Q.; Huang, H.; Maroney, M.J. The *Helicobacter pylori* HypA·UreE2 complex contains a novel high-affinity Ni(II)-binding site. *Biochemistry* **2018**, *57*, 2932–2942.
24. Mehta, N.; Olson, J.W.; Maier, R.J. Characterization of *Helicobacter pylori* nickel metabolism accessory proteins needed for maturation of both urease and hydrogenase. *J. Bacteriol.* **2003**, *185*, 726–734.
25. Leach, M.R.; Sandal, S.; Sun, H.; Zamble, D.B. Metal binding activity of the *Escherichia coli* hydrogenase maturation factor HypB. *Biochemistry* **2005**, *44*, 12229–12238.
26. Dias, A. V.; Mulvihill, C.M.; Leach, M.R.; Pickering, I.J.; George, G.N.; Zamble, D.B. Structural and biological analysis of the metal sites of *Escherichia coli* hydrogenase accessory protein HypB. *Biochemistry* **2008**, *47*, 11981–11991.
27. Chan Chung, K.C.; Cao, L.; Dias, A. V; Pickering, I.J.; George, G.N.; Zamble, D.B. A high-affinity metal-binding peptide from Escherichia coli HypB. *J. Am. Chem. Soc.* **2008**, *130*, 14056–14057.
28. Xia, W.; Li, H.; Yang, X.; Wong, K.B.; Sun, H. Metallo-GTPase HypB from *Helicobacter pylori* and its interaction with nickel chaperone protein HypA. *J. Biol. Chem.* **2012**, *287*, 6753–6763.
29. Sydor, A.M.; Lebrette, H.; Ariyakumaran, R.; Cavazza, C.; Zamble, D.B. Relationship between Ni(II) and Zn(II) coordination and nucleotide binding by the *Helicobacter pylori* [NiFe]-hydrogenase and urease maturation factor HypB. *J. Biol. Chem.* **2014**, *289*, 3828–3841.
30. Sydor, A.M.; Liu, J.; Zamble, D.B. Effects of metal on the biochemical properties of *Helicobacter pylori* HypB, a maturation factor of [NiFe]-hydrogenase and urease. *J. Bacteriol.* **2011**, *193*, 1359–1368.
31. Fu, C.; Olson, J.W.; Maier, R.J. HypB protein of *Bradyrhizobium japonicum* is a metal-binding GTPase capable of binding 18 divalent nickel ions per dimer. *Proc. Natl. Acad. Sci.* **1995**, *92*, 2333–2337.
32. Kaluarachchi, H.; Sutherland, D.E.; Young, A.; Pickering, I.J.; Stillman, M.J.; Zamble, D.B. The Ni(II)-binding properties of the metallochaperone SlyD. *J. Am. Chem. Soc.* **2009**, *131*, 18489–18500.
33. Cheng, T.; Li, H.; Xia, W.; Sun, H. Multifaceted SlyD from *Helicobacter pylori*: implication in [NiFe] hydrogenase maturation. *JBIC J. Biol. Inorg. Chem.* **2012**, *17*, 331–343.
34. Kaluarachchi, H.; Altenstein, M.; Sugumar, S.R.; Balbach, J.; Zamble, D.B.; Haupt, C. Nickel binding and [NiFe]-hydrogenase maturation by the metallochaperone SlyD with a single metal-binding site in *Escherichia coli*. *J. Mol. Biol.* **2012**, *417*, 28–35.
35. Lee, M.H.; Pankratz, H.S.; Wang, S.; Scott, R.A.; Finnegan, M.G.; Johnson, M.K.; Ippolito, J.A.; Christianson, D.W.; Hausinger, R.P. Purification and characterization of *Klebsiella aerogenes* UreE protein: a nickel-binding protein that functions in urease metallocenter assembly. *Protein Sci.* **1993**, *2*, 1042–1052.
36. Bellucci, M.; Zambelli, B.; Musiani, F.; Turano, P.; Ciurli, S. *Helicobacter pylori* UreE, a urease accessory protein: specific Ni^{2+}- and Zn^{2+}-binding properties and interaction with its cognate UreG. *Biochem. J.* **2009**, *422*, 91–100.
37. Banaszak, K.; Martin-Diaconescu, V.; Bellucci, M.; Zambelli, B.; Rypniewski, W.; Maroney, M.J.; Ciurli, S. Crystallographic and X-ray absorption spectroscopic characterization of *Helicobacter pylori* UreE bound to Ni^{2+} and Zn^{2+} reveals a role for the disordered C-terminal arm in metal trafficking. *Biochem. J.* **2012**, *441*, 1017–1035.
38. Stola, M.; Musiani, F.; Mangani, S.; Turano, P.; Safarov, N.; Zambelli, B.; Ciurli, S. The nickel site of *Bacillus pasteurii* UreE, a urease metallo-chaperone, as revealed by metal-binding studies and X-ray absorption spectroscopy. *Biochemistry* **2006**, *45*, 6495–6509.
39. Ciurli, S.; Safarov, N.; Miletti, S.; Dikiy, A.; Christensen, S.K.; Kornetzky, K.; Bryant, D.A.; Vandenberghe, I.; Devreese, B.; Samyn, B.; Remaut, H.; van Beeuman, J. Molecular characterization of *Bacillus pasteurii* UreE, a metal-binding chaperone for the assembly of the urease active site. *JBIC J. Biol. Inorg. Chem.* **2002**, *7*, 623–631.

40. Colpas, G.J.; Brayman, T.G.; Ming, L.J.; Hausinger, R.P. Identification of metal-binding residues in the *Klebsiella aerogenes* urease nickel metallochaperone, UreE. *Biochemistry* **1999**, *38*, 4078–4088.
41. Grossoehme, N.E.; Mulrooney, S.B.; Hausinger, R.P.; Wilcox, D.E. Thermodynamics of Ni^{2+}, Cu^{2+}, and Zn^{2+} binding to the urease metallochaperone UreE. *Biochemistry* **2007**, *46*, 10506–10516.
